# The Structure of the *Arabidopsis* PEX4-PEX22 Peroxin Complex—Insights Into Ubiquitination at the Peroxisomal Membrane

**DOI:** 10.3389/fcell.2022.838923

**Published:** 2022-02-18

**Authors:** Melissa S. Traver, Sarah E. Bradford, Jose Luis Olmos, Zachary J. Wright, Mitchell D. Miller, Weijun Xu, George N. Phillips, Bonnie Bartel

**Affiliations:** ^1^ Department of Biosciences, Rice University, Houston, TX, United States; ^2^ Department of Chemistry, Rice University, Houston, TX, United States

**Keywords:** *Arabidopis thaliana*, peroxin, ubiquitin conjugating enzyme (E2), organelle tether, X-ray crystal analysis, peroxisome, ubiquitination

## Abstract

Peroxisomes are eukaryotic organelles that sequester critical oxidative reactions and process the resulting reactive oxygen species into less toxic byproducts. Peroxisome function and formation are coordinated by peroxins (PEX proteins) that guide peroxisome biogenesis and division and shuttle proteins into the lumen and membrane of the organelle. Despite the importance of peroxins in plant metabolism and development, no plant peroxin structures have been reported. Here we report the X-ray crystal structure of the PEX4-PEX22 peroxin complex from the reference plant *Arabidopsis thaliana*. PEX4 is a ubiquitin-conjugating enzyme (UBC) that ubiquitinates proteins associated with the peroxisomal membrane, and PEX22 is a peroxisomal membrane protein that anchors PEX4 to the peroxisome and facilitates PEX4 activity. We co-expressed *Arabidopsis* PEX4 as a translational fusion with the soluble PEX4-interacting domain of PEX22 in *E. coli*. The fusion was linked *via* a protease recognition site, allowing us to separate PEX4 and PEX22 following purification and solve the structure of the complex. We compared the structure of the PEX4-PEX22 complex to the previously published structures of yeast orthologs. *Arabidopsis* PEX4 displays the typical UBC structure expected from its sequence. Although *Arabidopsis* PEX22 lacks notable sequence identity to yeast PEX22, it maintains a similar Rossmann fold-like structure. Several salt bridges are positioned to contribute to the specificity of PEX22 for PEX4 versus other *Arabidopsis* UBCs, and the long unstructured PEX22 tether would allow PEX4-mediated ubiquitination of distant peroxisomal membrane targets without dissociation from PEX22. The *Arabidopsis* PEX4-PEX22 structure also revealed that the residue altered in *pex4-1* (P123L), a mutant previously isolated *via* a forward-genetic screen for peroxisomal dysfunction, is near the active site cysteine of PEX4. We demonstrated *in vitro* UBC activity for the PEX4-PEX22 complex and found that the pex4-1 enzyme has reduced *in vitro* ubiquitin-conjugating activity and altered specificity compared to PEX4. Our findings illuminate the role of PEX4 and PEX22 in peroxisome structure and function and provide tools for future exploration of ubiquitination at the peroxisome surface.

## Introduction

Peroxisomes are membrane-bound organelles that are essential for life in almost all multicellular eukaryotes because they house critical metabolism, including the β-oxidation of fatty acids and the breakdown of hydrogen peroxide and other reactive oxygen species. In plants, peroxisomes are the sole site of fatty acid β-oxidation and also participate in photorespiration, biosynthesis of several phytohormones, co-factor biosynthesis, and diverse secondary metabolic pathways (reviewed in Reumann and Bartel, 2016).

Peroxisomes import fully-folded and even oligomeric proteins (McNew and Goodman, 1994; Lee et al., 1997) *via* the coordinated action of a group of peroxins (PEX proteins). This import is mediated by shuttling cargo receptors that accompany proteins containing a peroxisome-targeting signal (PTS) to the organelle. For example, PEX5 binds to cytosolic cargo proteins that have a C-terminal PTS1 and guides them to a docking complex at the peroxisomal membrane, where PEX5 inserts into the membrane to deliver the cargo into the organelle (reviewed in Kao et al., 2018). Returning PEX5 to the cytosol for additional rounds of import requires ubiquitination of a cysteine residue near the PEX5 N-terminus mediated by a complex of RING-type ubiquitin-protein ligases: PEX2, PEX10, and PEX12 (Kragt et al., 2005; Carvalho et al., 2007; Grou et al., 2008; Platta et al., 2009). In metazoans, these RING peroxins recruit a cytosolic ubiquitin-conjugating enzyme (UBC) (Grou et al., 2008). In contrast, yeast RING peroxins collaborate with a dedicated UBC, Pex4, or a cytosolic UBC, Ubc4, to ubiquitinate Pex5 for recycling or degradation, respectively (Kragt et al., 2005; Platta et al., 2007; Williams et al., 2008). Yeast Pex4 is anchored to the outside of the peroxisomal membrane by binding to Pex22 (Koller et al., 1999), a rapidly-evolving protein with an N-terminal transmembrane domain inserted in the peroxisomal membrane and a C-terminal cytosolic Pex4-binding domain. Pex22 is required for Pex4 function beyond its role as a membrane anchor (Williams et al., 2012; El Magraoui et al., 2014). For example, in the presence of Pex22, *Ogataea angusta* (previously known as *Hansenula polymorpha*) Pex4 adopts an active conformation and is able to build polyubiquitin chains *in vitro* (Groves et al., 2018).

Like yeast, plants have a dedicated peroxisomal UBC, PEX4, that is tethered to peroxisomes *via* a membrane-bound peroxin, PEX22 (Zolman et al., 2005). Although no validated null alleles of either PEX4 or PEX22 are present in publicly available T-DNA collections (Alonso et al., 2003), two *Arabidopsis thaliana pex4* mutants have emerged from forward-genetic screens for peroxisome dysfunction (Zolman et al., 2000, 2005; Kao and Bartel, 2015). The *pex4-1* P123L missense allele confers peroxisome-defective phenotypes that are exacerbated in combination with the *pex22-1* mutant, a T-DNA insertion in the 5′-UTR of *PEX22* (Zolman et al., 2005). The *pex4-2* mutant carries an intronic mutation that reduces accumulation of PEX4 protein and displays similar but less severe defects than *pex4-1* (Kao and Bartel, 2015). Thus, both *pex4-1* and *pex4-2* are partial-loss-of-function alleles. Several peroxin mutations confer embryo lethality in *A. thaliana*, including null alleles of any of the three RING peroxins that guide PEX4 to ubiquitinate PEX5 (Schumann et al., 2003; Sparkes et al., 2003; Fan et al., 2005; Prestele et al., 2010), but it is unknown whether PEX4 or PEX22 are essential for embryogenesis. Additionally, whereas many of the 37 potential UBCs in *A. thaliana* have been experimentally validated (Bachmair et al., 2001; Kraft et al., 2005), ubiquitination by PEX4 has not been directly demonstrated. In contrast to many UBCs, which are encoded by closely related genes in Arabidopsis, phylogenetic analysis places PEX4 as the sole member of a UBC subfamily (Kraft et al., 2005). This observation, along with the characteristic peroxisome-defective phenotypes of *pex4* mutants (Zolman et al., 2005; Kao and Bartel, 2015), implies that PEX4 acts non-redundantly and that PEX22 binds specifically to PEX4 among all UBCs. The structural basis for this specificity is unknown.

The three RING peroxins function in a complex (El Magraoui et al., 2012). Partial loss-of-function alleles of any of the *A. thaliana* RING peroxins, PEX2, PEX10, and PEX12, can destabilize other members of the complex (Burkhart et al., 2014; Kao et al., 2016). Unexpectedly, reducing PEX4 function *via* the *pex4-1* or *pex4-2* mutations partially restores RING complex stability and peroxisome function in the *A. thaliana pex12-1* mutant (Kao et al., 2016). These data suggest that the lysine residue provided by the pex12-1 E171K mutation serves as an ectopic PEX4 ubiquitination site, leading to pex12-1 degradation and destabilization of the RING complex when PEX4 is functional (Kao et al., 2016). Moreover, certain lumenal proteins are stabilized in a *pex4-1 pex22-1* double mutant (Lingard et al., 2009). Thus, it is possible that PEX4 participates in ubiquitination of peroxisomal proteins beyond its established substrate, PEX5.

**FIGURE 1:**
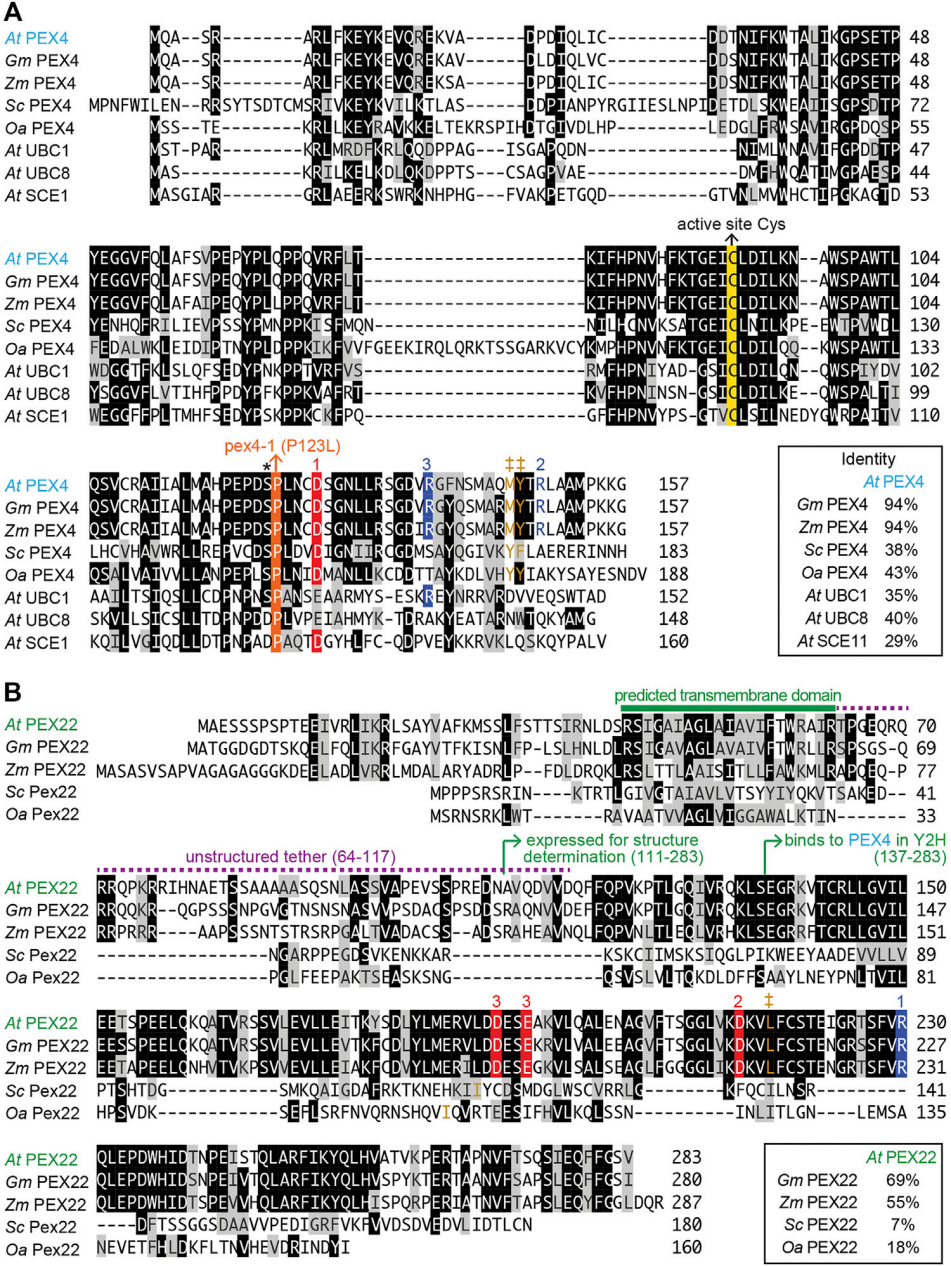
Alignment of *Arabidopsis* PEX4 and PEX22 with homologs from *Arabidopsis* and other organisms. Ar*abidopsis thaliana* (*At*) PEX4 is highly conserved in plants [*Glycine max* (*Gm*), XP_003522698.1; *Zea mays* (*Zm*), NP_001130714.1] and similar to yeast Pex4 [*Saccharomyces cerevisiae* (*Sc*), P29340.1; *Ogataea angusta* (*Oa*), O60015.1] and other *A. thaliana* conjugating enzymes (*At* UBC1, OAP18713.1; *At* UBC8, NP_001190447.1; *At* SCE1, Q42551.1) **(A)**, whereas *A. thaliana* PEX22 (*At* PEX22) is somewhat conserved in plants (*Gm*, XP_003543380.1; Zm, NP_001358459.1) but is highly diverged from yeast Pex22 (*Sc*; KZV13408.1; *Oa*, ABD37672.1) **(B)**. Proteins were aligned using the Clustal W method of the Megalign program (DNAStar). Identical residues in at least four **(A)** or three **(B)** sequences are in black or colored boxes; chemically similar residues are in gray. The active site cysteine (yellow), the pex4-1 missense mutation (orange), and the “gateway” residue (asterisk; reviewed in Stewart et al., 2016) are highlighted in panel **A**. Numbers above sequences indicate acidic (red) or basic (blue) residues involved in three *At* PEX4-PEX22 salt bridges deduced from the crystal structure. Hydrophobic residues in analogous positions as Y172 in *Sc* Pex4, which is essential for *Sc* Pex4-Pex22 interaction (Williams et al., 2012), and interacting hydrophobic residues in PEX22 are shown in brown and marked with ‡.

Structural information is available for two yeast (*S. cerevisiae* and *O. angusta*) Pex4-Pex22 complexes (Williams et al., 2012; Groves et al., 2018). These studies reveal that yeast Pex4 binds to Pex22 *via* a binding site at the C-terminus of Pex4 that does not overlap with other known UBC-interaction surfaces (Williams et al., 2012; Groves et al., 2018). Whereas *A. thaliana* PEX4 is 38–42% identical to its yeast orthologs (Figure 1A) and was originally identified by homology to yeast Pex4 (Mullen et al., 2001), *A. thaliana* PEX22 lacks notable amino acid sequence similarity to the yeast proteins (Figure 1B), and was originally identified by its ability to bind to *A. thaliana* PEX4 in yeast two-hybrid experiments (Zolman et al., 2005).

To elucidate the basis of PEX4-PEX22 specificity, to better understand the nature of *pex4* mutations, and to begin to address the lack of plant peroxin structural information, we solved the crystal structure of *A. thaliana* PEX4 complexed with the C-terminal domain of PEX22. We analyzed this structure to illuminate the details of PEX4-PEX22 binding. Additionally, we demonstrated *in vitro* PEX4-PEX22 ubiquitin-conjugating activity and found that the protein encoded by the *pex4-1* missense allele displays altered ubiquitination specificity without markedly impacting PEX22 binding.

## Materials and Methods

### Plant Materials and Growth Conditions

Plants in the Col-0 accession of *Arabidopsis thaliana* and the *pex4-1* missense allele (Zolman et al., 2005) were grown at 22°C under constant light. Seeds were surface sterilized and sown on plant nutrient (PN) medium (Haughn and Somerville, 1986) solidified with 0.6% (w/v) agar and supplemented with 0.5% (w/v) sucrose. Seedlings were collected for immunoblotting or transferred to soil after 1–2 weeks for seed production.

### Construction of PEX4-PEX22 Expression Plasmids

We constructed a plasmid for expressing full-length *A. thaliana* PEX4 (At5g25760; also named UBC21) without a stop codon and fused to truncated PEX22 (At3g21865; residues 111-283) *via* a linker consisting of a PreScission protease (Genscript Z03092) cleavage site (LEVLFQ|GP, where “|” designates the cleavage site). The PEX4-PEX22 fusion was expressed in *E. coli* with an N-terminal His_6_-maltose-binding protein (MBP) tag that was connected *via* a linker consisting of a TEV protease cleavage site (ENLYFQ|S). *PEX4* and *PEX22* cDNA fragments were cloned into the pET-His6-MBP-TEV-LIC cloning vector (a gift from Scott Gradia; Addgene plasmid 29656). PCR amplification was performed on the pET vector using primers pET-F and pET-R (Supplementary Table S1). Primers PEX4-F and PEX4-R (Supplementary Table S1) were used to PCR amplify a *PEX4* cDNA (Zolman et al., 2005), and primers PEX22-F and PEX22-R (Supplementary Table S1) were used to PCR amplify a *PEX22* cDNA (Zolman et al., 2005). The PEX4, PEX22, and pET vector amplicons were combined using Gibson assembly (Gibson et al., 2009) with Gibson Assembly Master Mix (New England Biolabs E2611) to give plasmid His6-MBP-PEX4-PEX22, which was verified by sequencing.

To construct His6-MBP-pex4^P123L^-PEX22, Gibson assembly was used to combine the pET vector (amplified with pET-F and pET-R) with PCR products amplified from the His6-MBP-PEX4-PEX22 plasmid with primers PEX4-F paired with pex4-1-R and pex4-1-PEX22-F paired with PEX22-R (Supplementary Table S1).

### PEX4-PEX22 Protein Expression and Purification

Chemically competent BL21 Star (DE3) *E. coli* cells (Invitrogen C601003) were transformed with either the His_6_-MBP-PEX4-PEX22 or His_6_-MBP-pex4^P123L^-PEX22 expression plasmids. Transformed *E. coli* were selected on LB (Fisher BP1426) agar plates supplemented with kanamycin (50 μg/mL). Single colonies were used to inoculate 50 mL cultures of LB supplemented with kanamycin (50 μg/mL), and cultures were incubated overnight at 37°C with shaking. 3 mL of each overnight culture was used to inoculate 500 mL of Terrific Broth (Invitrogen 22711022) supplemented with kanamycin (50 μg/mL) and cultures were incubated at 37°C with shaking to an OD_600_ of 0.6–0.8. Cultures were then supplemented to 0.75 mM isopropyl β-D-1-thiogalactopyranoside (IPTG) and incubated 30°C overnight with shaking. Cells were harvested by centrifugation and stored at −80°C until lysis.

Partially thawed cell pellets were resuspended in 5 mL lysis buffer (25 mM HEPES pH 8, 300 mM NaCl, 5% glycerol, 10 mM imidazole, 1 mM Tris(2-carboxyethyl)phosphine hydrochloride (TCEP)) per gram of cell pellet and lysed by sonication on ice for 5 min (cycling 15 s on, 15 s off) or 10 min (cycling 1 s on, 1 s off). The lysate was then clarified by centrifugation (39,000 x *g*) and incubated for at least 1 h with 10 mL Ni-NTA Superflow resin (Qiagen 30410) equilibrated with lysis buffer at 4°C with rocking. After incubation, the resin was loaded onto a gravity-flow column and washed with 50 mL buffer A (25 mM HEPES pH 8, 300 mM NaCl, 5% glycerol, 1 mM TCEP) and then washed sequentially with 12.5 mL aliquots of a step gradient from 20 to 500 mM imidazole, using combinations of buffer A and buffer B (25 mM HEPES pH 8, 300 mM NaCl, 5% glycerol, 1 mM TCEP, 500 mM imidazole). The presence of eluted protein was assessed using SDS-PAGE and Coomassie staining (SimplyBlue SafeStain, Fisher LC6060). Fractions containing the target protein were concentrated to 2 mL using centrifugal concentrators (3,000 Da cutoff, Amicon Ultra-15, Millipore Sigma UFC900324) and subjected to cleavage with His-tagged PreScission protease (Genscript Z03092) alone or with His-tagged TEV protease, produced as described (Tropea et al., 2009). Using a ratio of 1 mg TEV protease and 500 units PreScission protease for every 50 mg of fusion protein, the reaction mixture was allowed to incubate overnight at 4°C on a rotary mixer then clarified by centrifugation (39,000 × *g*). TEV protease, PreScission protease, and N-terminal cleavage products containing His_6_ tags were removed by an additional purification with Ni-NTA Superflow resin equilibrated with buffer A. PEX4 or pex4-1 and PEX22^111−283^ were present in the flowthrough and in the first two to three washes with buffer A as confirmed by SDS-PAGE and Coomassie staining. Fractions containing PEX4 or pex4-1 and PEX22^111−283^ were concentrated, and PEX4-PEX22^111−283^ fractions selected for crystallization were further purified using anion-exchange resin HiTrap DEAE Sepharose FF (Cytiva 17515401) equilibrated with buffer 1 (25 mM HEPES pH 8, 5% glycerol, 1 mM TCEP). PEX4 and PEX22^111−283^ were present in the flowthrough as confirmed by SDS-PAGE and Coomassie staining.

### PEX4-PEX22 Protein Crystallization

Purified PEX4-PEX22^111−283^ was concentrated using centrifugal concentrators (10,000 Da cutoff, Amicon Ultra-0.5, Millipore Sigma) to 20 mg/mL in 20 mM Tris pH 7.5, 200 mM NaCl, 1 mM DTT for crystallization trials. A 1:1 ratio was assumed and a calculated extinction coefficient of 0.850 M^−1^ cm^−1^ for PEX4 and PEX22 (https://www.expasy.org) was used to estimate concentration. PEX4-PEX22^111−283^ crystallization conditions were established using a sitting drop vapor diffusion setup with a Mosquito LCP automated high-throughput nanoliter pipettor (SPT Labtech) and several commercial crystallization screening kits, including PEG Rx HT, Index HT (Hampton Research), Wizard Classic 1 and 2, 3 and 4 (Rigaku Reagents), Midas, and Morpheus (Molecular Dimensions). Crystal hits were identified *via* UV-excitation of aromatic amino acids using a JANSi UVEX instrument (SWISSCI). Crystals suitable for X-ray diffraction were grown at 20°C after mixing 200 nL of recombinant protein (20 mg/mL) with 200 nL of precipitant (100 mM NaCl, 100 mM Bis-Tris Propane pH 9.0, 25% (w/v) PEG 1,500) from the reservoir. Crystals were transferred to a cryobuffer consisting of reservoir buffer supplemented with 10% (v/v) glycerol, flash cooled in liquid nitrogen, and shipped using a dry shipping dewar (Taylor-Wharton) to Argonne National Laboratory’s Advanced Photon Source (Lemont, IL, United States) for diffraction data collection.

### Data Collection, Structural Determination, Refinement, and Analysis

PEX4-PEX22^111−283^ crystals diffracted to a resolution of 2.01 Å, and X-ray data were collected on the 23-ID-B beamline at Argonne National Laboratory’s Advanced Photon Source (Lemont, IL, United States) at a wavelength of 1.033 Å with an EIGER X 16M detector (DECTRIS Ltd.). Data were processed using the *autoPROC* toolbox (Vonrhein et al., 2011), which indexed and integrated the data with *XDS* (Kabsch, 2010) and scaled the data with *aimless* (Winn et al., 2011; Evans and Murshudov, 2013). Crystals belonged to the C-centered orthorhombic space group C222_1_, with unit cell lengths of 57.39, 100.48, and 112.10 Å. Initial phases were obtained by molecular replacement with Phaser-MR (McCoy et al., 2007; Liebschner et al., 2019) using the *O. angusta* Pex4-Pex22 complex structure (PDB ID: 5NKZ; PEX4: 43% identity) as a search model for PEX4, and an FFAS/SCRWL built poly-Ser model for PEX22 (Jaroszewski et al., 2005). This initial MR phasing solution was subjected to iterative density modification and poly-alanine auto-tracing with *shelxe* (Sheldrick, 2008; Thorn and Sheldrick, 2013), which improved the phases and built an initial poly-Ala model of PEX22. The *shelxe* poly-Ala model was rebuilt and side-chains docked using *ARP/wARP* (Langer et al., 2008). Autotracing with *shelxe* and *ARP/wARP* failed to build the PEX22 C-terminal helix. However, MR runs using *ab initio* models generated with AWSEM-Suite (Jin et al., 2020) correctly placed the PEX22 C-terminal helix, which was merged with the ARP/wARP model. Subsequent model building and refinement were performed with Coot (Emsley et al., 2010) and phenix.refine (Liebschner et al., 2019). Data processing and refinement software were compiled and supported by the SBGrid Consortium (Morin et al., 2013). Structures were viewed and analyzed using a collaborative 3-D visualization system (Yennamalli et al., 2014), and structural figures were prepared using ChimeraX (Goddard et al., 2018; Pettersen et al., 2021; https://www.rbvi.ucsf.edu/chimerax). Data processing and refinement statistics are shown in Table 1. PDB-wide structure comparisons were performed with Dali (Holm, 2020) using the PDB90 reference dataset and a Z-value cutoff of >6.0. Root-mean-square deviation (RMSD) values were calculated with Dali (over the alignment length for whole subunits) or the MatchMaker function in ChimeraX (for the interfacing residue regions).

**TABLE 1:** Data collection and refinement statistics for *Arabidopsis* PEX4-PEX22 structure.

PDB ID	6XOD
Space group	C 2 2 2_1_
Unit cell lengths (Å)	a = 57.39 b = 100.48 c = 112.10
Temperature (K)	100
Wavelength (Å)	1.033
Data collection statistics	
Resolution range (Å)	56.0–2.01 (2.04–2.01)^a^
Number of observations	144,846 (6499)
Number of unique reflections	22014 (1079)
Completeness (%)	99.9 (100.0)
R_merge_ ^b^	0.096 (1.56)
R_meas_ ^c^	0.104 (1.71)
Redundancy	6.6 (6.0)
Mean I/σ	10.5 (1.1)
CC_1/2_ ^d^	0.998 (0.464)
Wilson B-factor	43.6
Refinement statistics	
Resolution range (Å)	49.8–2.01 (2.10–2.01)
R_cryst_ ^e^	0.194 (0.278)
R_free_ ^f^	0.237 (0.320)
RMSD bonds (Å)	0.004
RMSD angles (°)	1.0
Average B factors (Å^2^)	
Protein	56.5
Water	55.1
Number of protein non-H atoms	2562
Number of water molecules	131
Ramachandran favored (%)	97.2
Ramachandran allowed (%)	2.5
Ramachandran outliers (%)	0.3
Clashscore	1.8

a Values in parentheses are for the highest-resolution shell.

b R_merge_ = Σ_h_Σ_i_|I_i_(h) - < I(h)>|/Σ_h_Σ_i_I_i_(h), where I_i_(h) is the intensity of an individual measurement of the reflection and <I(h)> is the mean intensity of the reflection.

c R_meas_ = Σ_h_(n_h_/(n_h_-1) Σ_i_|I_i_(h) - < I(h)>|/Σ_h_Σ_i_I_i_(h), where n_h_ denotes the redundancy.

d CC_1/2_ = ∑ (x—<x>)(y—<x>)/[∑(x—<x>)^2^Σ(y—<y>)^2^]^1/2^

e R_cryst_ = Σ_h_||F_obs_|-|F_calc_||/Σ_h_|F_obs_|, where F_obs_ and F_calc_ are the observed and calculated structure factor amplitudes, respectively.

f R_free_ was calculated as R_cryst_ using 5% of the randomly selected unique reflections that were omitted from structure refinement.

### *In vitro* Ubiquitination Assays

*In vitro* ubiquitination reactions were performed at 30°C in 1X ubiquitination reaction buffer (Boston Biochem SK-10) at a total volume of 50 μL and contained: 33 µM ubiquitin (either Boston Biochem *A. thaliana* wild-type ubiquitin, U-100At; R&D Systems human K48R ubiquitin, UM-K48R-01M; or R&D Systems human K48-only ubiquitin, UM-K480-01M), 0.1 μM human E1 (His_6_-UBE1; Boston Biochem E-304), and 5 μM purified *A. thaliana* PEX4- or pex4^P123L^-PEX22^111−283^. Reactions were initiated by the addition of Mg-ATP to 1 mM (Boston Biochem, SK-10) and quenched with 1X E1 Stop Buffer (Boston Biochem, SK-10). Proteins were visualized using SDS-PAGE and SimplyBlue SafeStain or immunoblotting.

### Immunoblot Analysis

Two volumes of sample buffer (500 mM Tris pH 8.0, 4% (w/v) lithium dodecyl sulfate, 1 mM EDTA, 20% (w/v) glycerol, 22 µM Coomassie blue G250, 16.6 μM mM phenol red, 50 µM dithiothreitol (DTT)) were added to *in vitro* ubiquitination samples, which were then heated at 100°C for 5 min. Equal volumes of sample (3 µL for immunoblots; 20 µL for Coomassie-stained gels) and pre-stained markers (New England Biolabs, P7712 or P7719) were loaded on Bolt 10% (w/v) Bis-Tris gels (Invitrogen). Gels were electrophoresed in 50 mM MES-SDS running buffer (50 mM MES hydrate, 50 mM Tris base, 3.5 mM SDS, 1.27 mM EDTA). Proteins were transferred from the gel to Hybond-ECL nitrocellulose membranes (Amersham, Protran Premium 0.45 μm NC 10600003) using the standard setting on a GenScript eBlot L1 transfer system (GenScript, L00686). Following transfer, membranes were air-dried at room temperature (RT) for 1 h followed by 1 h blocking with Intercept Blocking Buffer (LI-COR, 927-60001) at RT with rocking. After blocking, membranes were incubated overnight with rocking at 4°C with various primary antibodies diluted in 8% (w/v) Carnation instant non-fat dry milk in TBST (20 mM Tris pH 7.5, 150 mM NaCl, 0.1% (v/v) Tween-20). Membranes were then incubated 1–2 h with secondary antibody with rocking at RT. Primary antibodies used were rabbit anti-PEX4 (1:100; Kao and Bartel, 2015) and mouse anti-ubiquitin (P4D1, 1:300; Santa Cruz sc-8017). Secondary antibodies used were horseradish peroxidase (HRP)-linked goat anti-rabbit antibody (1:5,000; GenScript A00098) and IRDye 800CW-linked goat anti-mouse secondary antibodies (1:10,000, LI-COR 926-32210). Following secondary antibody incubation, membranes were imaged with (HRP-linked secondary antibodies) or without (IRDye secondary antibodies) WesternSure Premium Chemiluminescent substrate (Fisher, 50-489–552) using an Odyssey Fc imaging system (LI-COR, 2801-02). Quantification was performed using Image Studio software (LI-COR, version 5.2). Ubiquitination activity rates were separately calculated for three replicates using the slope value of a linear trendline. Statistical comparison of rates between wild type and mutant purified proteins was performed using Graphpad Prism (version 9.3.0).

Fractionation of extracts from 6-day-old dark-grown seedlings was conducted as previously described (Kao and Bartel, 2015). Proteins were separated on Bolt 10% (w/v) Bis-Tris gels and transferred to membranes as described above. Membranes were probed sequentially (without stripping) with rabbit anti-PEX4 (1:100), rabbit anti-PEX5 (1:100; Zolman and Bartel, 2004), mouse anti-HSC70 (1:50,000), rabbit anti-PEX14 (1:10,000, Agrisera AS08 372), mouse anti-mitochondrial ATP synthase α subunit (1:2,000; Mito-Science MS507), and rabbit anti-isocitrate lyase (1:1,000; Maeshima et al., 1988) and processed as described above.

### *In vitro* PEX4-PEX22 Interaction Assay

Purified His_6_-MBP-PEX4-PEX22 and His_6_-MBP-pex4^P123L^-PEX22 were cut with PreScission protease as described above. The reaction was incubated for 1 h with Ni-NTA Superflow resin equilibrated with buffer A with rocking at 4°C, washed four times with buffer A, then washed four times with buffer B. Resin was separated from wash fractions *via* low-speed centrifugation. Samples from each fraction were analyzed *via* SDS-PAGE and Coomassie staining.

## Results

### PEX4-PEX22 Expression and Purification

**FIGURE 2:**
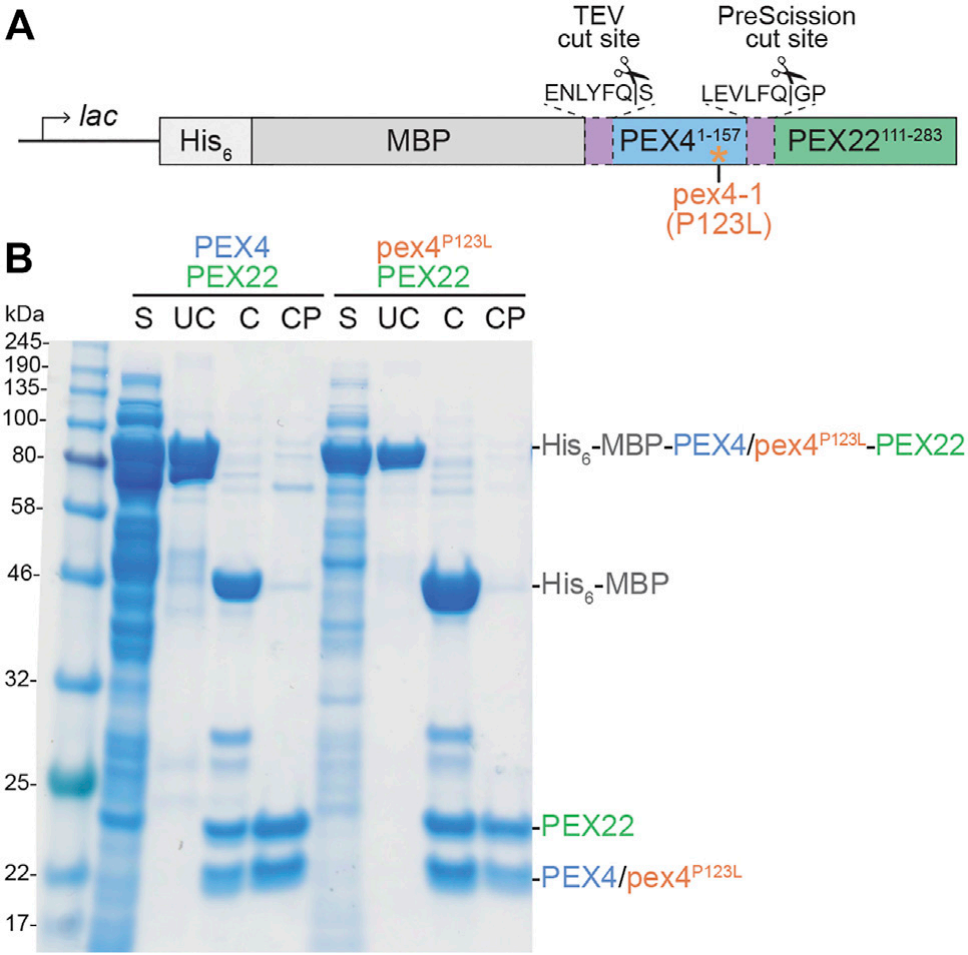
Expression and purification of *Arabidopsis* PEX4/pex4^P123L^-PEX22^111−283^ complexes. **(A)**. Schematic of constructs used to express full-length PEX4 and pex4-1 (pex4^P123L^) with PEX22 (amino acids 111-283). His_6_-MBP-PEX4/pex4^P123L^-PEX22^111−283^ fusions were expressed in *E. coli* following IPTG induction of the *lac* promoter. His_6_ was included as a purification tag, maltose binding protein (MBP) was included as a solubility tag, and unique protease sites separated MBP and PEX4/pex4^P123L^ (TEV cut site) and PEX4/pex4^P123L^ and PEX22^111−283^ (PreScission cut site). **(B)**. PEX4/pex4^P123L^ and PEX22^111−283^ purification. After expression in *E. coli*, cells were lysed and the full-length uncleaved (UC) protein (81 kDa) was purified from the soluble fraction (S). Purified protein was cleaved with TEV and PreScission proteases (C) and separated from His_6_-MBP (43 kDa) and proteases to give cleaved and purified (CP) PEX4/pex4^P123L^ (18 kDa) and PEX22^111−283^ (20 kDa).

To understand the specificity of plant PEX4-PEX22 interactions and to gain mechanistic insight into the defects conferred by the *pex4-1* missense allele, we determined the structure of the *A. thaliana* PEX4-PEX22 complex. *S. cerevisiae* Pex4 is inactive without Pex22 (Williams et al., 2012), and prior efforts to heterologously express *A. thaliana* PEX4 in *E. coli* or insect cells yielded insoluble enzyme (Kraft et al., 2005). Therefore, we co-expressed PEX4 and PEX22^111−283^ in *E. coli* as a translational fusion linked by a synthetic PreScission protease cleavage site (Figure 2A). The N-terminal region of *A. thaliana* PEX22 includes a predicted transmembrane domain (Figure 1B) and is dispensable for PEX4 binding in yeast two-hybrid assays (Zolman et al., 2005), thus we did not include the N-terminal 110 amino acids of PEX22 in the construct. We expressed PEX4 at the N-terminus of this PEX22 region because the C-terminus of Pex4 is only ∼21 Å from the N-terminus of Pex22 (lacking the transmembrane domain) in the yeast co-crystal structures (Williams et al., 2012, PDB ID 2Y9M; Groves et al., 2018, PDB ID 5NKZ). We expressed this *A. thaliana* PEX4-PEX22^111−283^ fusion with N-terminal His_6_ and maltose-binding protein (MBP) tags to facilitate purification and solubility, respectively. His_6_-MBP-PEX4-PEX22^111−283^ was soluble following expression in *E. coli*, and we purified the fusion protein using nickel chromatography, cleaved the fusion with TEV and PreScission proteases to separate His_6_-MBP, PEX4, and PEX22^111−283^, and removed the His_6_-MBP and His-tagged-proteases by collecting PEX4-PEX22^111−283^ from the flow-through of second round of nickel chromatography (Figure 2B). Ion-exchange chromatography of the PEX4-PEX22^111−283^ complex yielded protein suitable for crystallization.

### Crystal Structure of the *Arabidopsis* PEX4-PEX22 Complex

**FIGURE 3:**
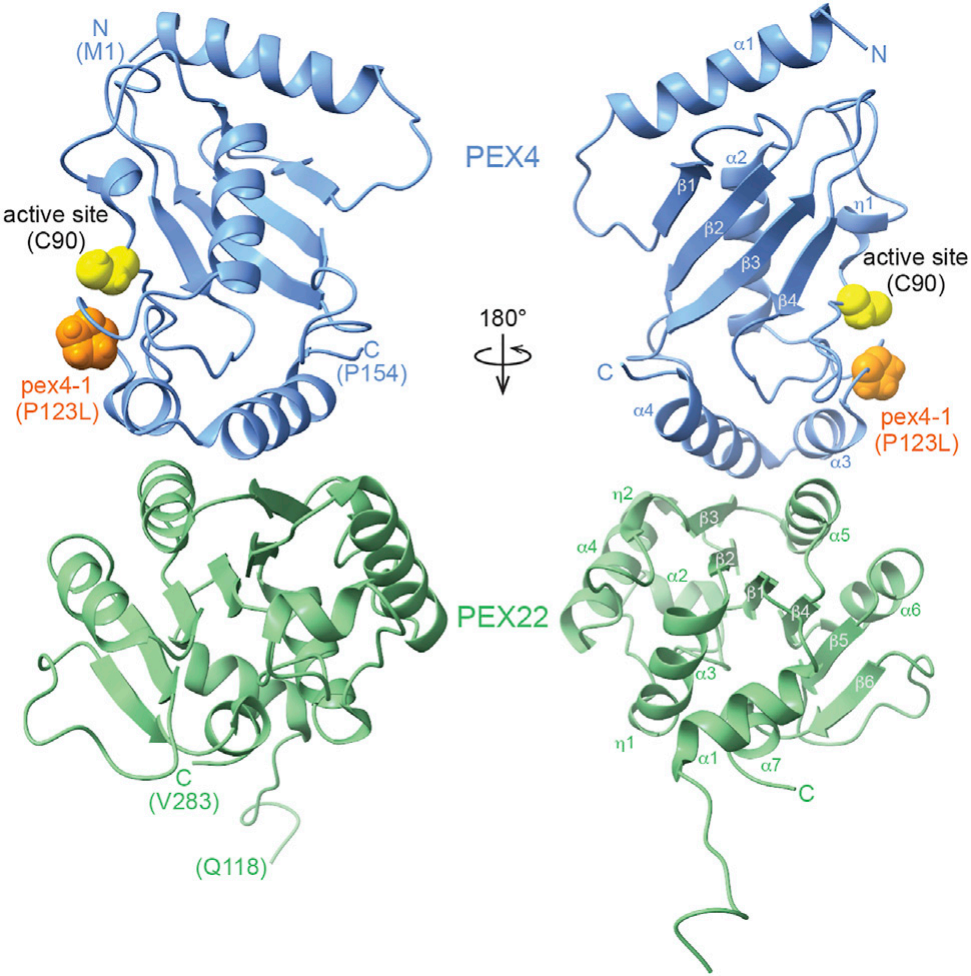
X-ray crystal structure of the *Arabidopsis thaliana* PEX4-PEX22^111−283^ complex. The ribbon diagram illustrates that PEX4 (blue) α-helices 3 and 4 interact with a surface of PEX22 (green) formed by a PEX22 3_10_ helix (η2), β-strand 3, and α-helix 5. The PEX4 active site cysteine (C90) is highlighted in yellow and is positioned between the PEX4 3_10_ helix (η1) and the loop connecting PEX4 α-helix 2 (the “crossover” helix) and α-helix 3. The proline residue mutated in *pex4-1* (P123L) is situated in this loop and is highlighted in orange.

We crystallized and solved the structure of the *A. thaliana* PEX4-PEX22^111−283^ complex at 2.01 Å resolution (PDB ID 6XOD; Table 1, Figure 3). The final model included residues 1-154 of PEX4, residues 118-283 of PEX22^111−283^, and 131 water molecules. One residue following the TEV protease cleavage site (0 at the N-terminus), three PEX4 C-terminal residues (155-157), and six C-terminal PreScission cleavage site residues (158-163) were disordered and were not modeled (Supplementary Figure S1). Similarly, two N-terminal PreScission cleavage site residues and seven N-terminal residues (111-117) of PEX22^111−283^ were disordered and were not modeled. The model includes a single Ramachandran outlier (PEX22 Pro^122^) that is part of the N-terminal linker region and can be explained by poor density.

**FIGURE 4:**
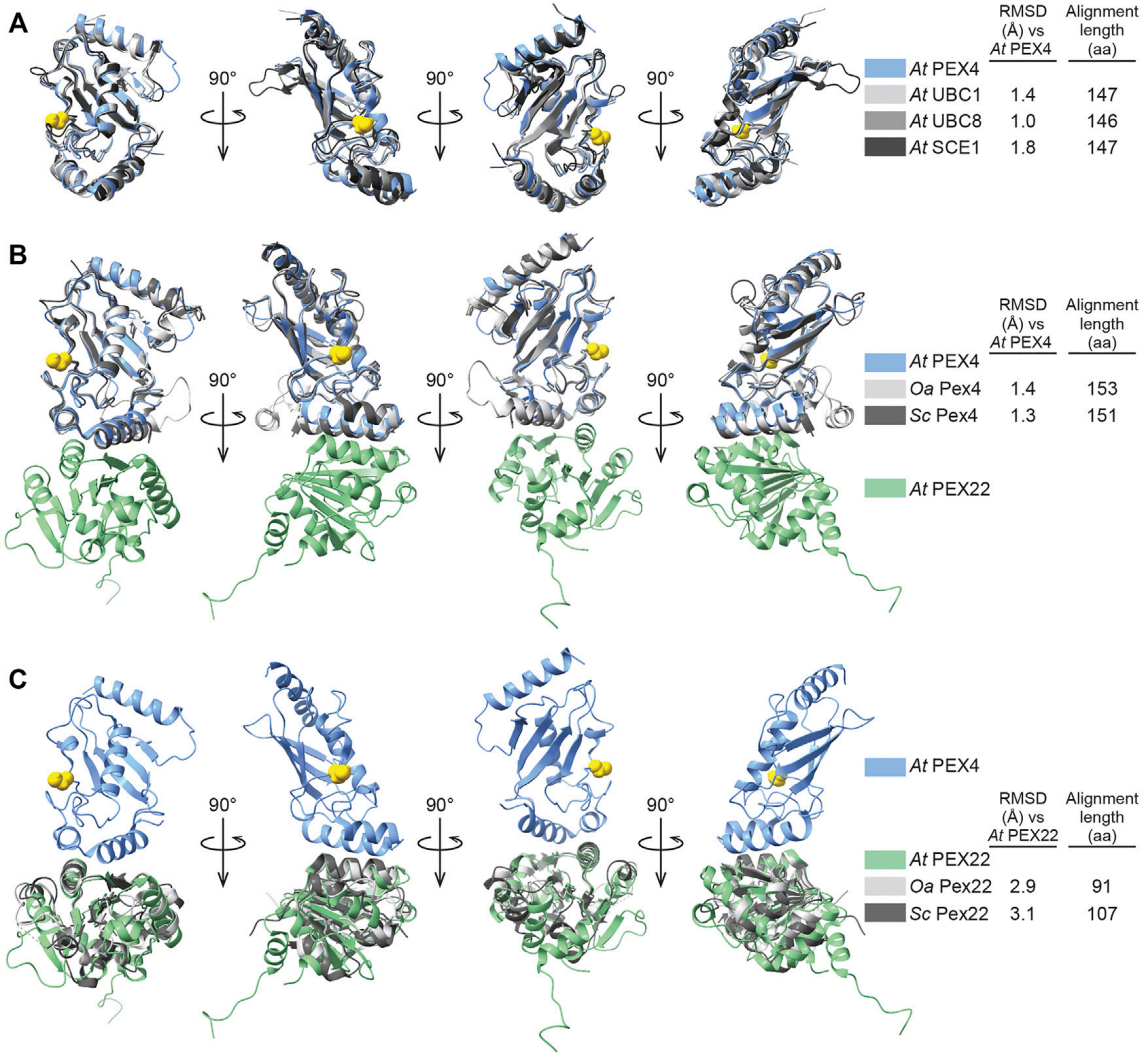
Comparison of *Arabidopsis* PEX4 and PEX22 with similar proteins in *Arabidopsis* and yeast. **(A)**. Aligned X-ray crystal structures of *Arabidopsis thaliana* (*At*) PEX4 with paralogs: ubiquitin-conjugating enzymes *At* UBC1 (PDB 2AAK) and *At* UBC8 (PDB 4X57), and the SUMO-conjugating enzyme *At* SCE1 (PDB 6GV3). The PEX4 active-site cysteine is highlighted in yellow. Root mean square deviation (RMSD) values and alignment lengths were computed by using pairwise Dali alignments (Holm, 2020). **(B,C)**. X-ray crystal structures of the *Arabidopsis thaliana* (*At*) PEX4-PEX22 complex aligned with orthologs from *Saccharomyces cerevisiae* (*Sc*, PDB 2Y9M) and *Ogataea angusta* (*Oa*, previously known as *Hansenula polymorpha*; PDB 5NKZ). Structures were aligned to PEX4 **(B)** or PEX22 **(C)**. The PEX4 active-site cysteine is highlighted in yellow. RMSD values and alignment lengths were computed by using pairwise Dali alignments (Holm, 2020).

PEX4 adopts a canonical UBC fold (reviewed in Stewart et al., 2016), with four α-helices, four β-strands, and the active site cysteine between β-strand 4 and α-helix 2 (Figure 3; Supplementary Figure S1). The active site cysteine (C90) is in a cleft bordered by the PEX4 3_10_ helix (η1) and the loop region between α-helices 2 and 3. α-helix 2 is the “crossover” helix that extends across the antiparallel β-sheet formed by the four β-strands (Figure 3). The PEX4 structure, including the active site region, aligns closely (1.0–1.8 Å RMSD) with structures of ubiquitin- and SUMO-conjugating enzymes from *A. thaliana* (Figure 4A). Similarly, aligning the *A. thaliana* PEX4 structure with Pex4 structures from *S. cerevisiae* and *O. angusta* revealed close similarity (1.3–1.4 Å RMSD), apart from the insertion found on the backside (relative to the active site cysteine) of the *O. angusta* enzyme (Figure 4B). A Dali (Holm, 2020) structural similarity search of PEX4 to the PDB90 dataset (the subset of the PDB with ≤90% identity) revealed numerous similar structures. As expected, most of the close matches (Z-values greater than 6.0; 80 structures) were UBC enzymes from a variety of eukaryotes, which displayed 22–44% sequence identity with *At* PEX4. The closest match was *Oa* Pex4 (44% identity, Z = 19.4), and *Sc* Pex4 was also detected (38% identity, Z = 22). Most proteins displaying between 10 and 20% identity with PEX4 were annotated as having a UBC-related domain. For example, PEX4-related proteins also included Ubiquitin E2 variant (UEV)-domains (a ubiquitin-binding domain that lacks the two C-terminal α-helices of the UBC domain; Sundquist et al., 2004) or RWD-domains (a domain found in RING fingers, WD-domain-containing-proteins, and DEAD-like helicases; Doerks et al., 2002).

We found that *A. thaliana* PEX22^111−283^ folds as a single domain consisting of a parallel six-stranded β-sheet flanked by α-helices on both sides (Figure 3). Comparing PEX22 to the PDB90 dataset using Dali (Holm, 2020) revealed 344 structures with Z-values greater than 6.0. The sequence identities for PEX22 matches were lower than for PEX4, ranging from 3 to 16%. Although not among the top matches, this list did include both *Sc* Pex22 (15% identity, Z = 8) and *Oa* Pex22 (13% identity, Z = 6.9) along with a variety of eukaryotic and bacterial enzymes. A topology plot for PEX22 (Supplementary Figure S2; Laskowski et al., 2018) revealed a pattern of alternating β-strands and α-helices similar to the Rossmann tertiary fold motif, with the exception of a loop rather than an α-helix joining β-strands 5 and 6 in PEX22 (Figure 3; Supplementary Figure S2). A Rossmann fold is found in about 20% of structures in the PDB, often as a nucleotide binding domain (reviewed in Shin and Kihara, 2019). This fold is the likely origin of the broad homology matches to PEX22. Indeed, a Rossmann fold resemblance was previously noted for yeast Pex22 (Williams et al., 2012).

We aligned the *A. thaliana* PEX22^111−283^ structure with the two yeast Pex22 structures (Figure 4C). In parallel with reduced amino acid sequence similarity (Figure 1B), the PEX22^111−283^ structure aligns less precisely to those of its homologs (∼3 Å RMSD over a 91-107 aa alignment) than PEX4. We observed considerable differences at both the N- and C-terminus of the structures. For example, α-helix 1 of *At* PEX22 is not found in the yeast Pex22 proteins, which both have a β-strand as the first secondary structural element (Supplementary Figure S2). Moreover, the *At* PEX22 β-sheet is comprised of six β-strands (Figure 3), whereas the yeast proteins have five β-strands (Supplementary Figure S2). Notably, the region of highest similarity appeared to be the interface between PEX4 and PEX22 (Figure 4C). Indeed, the Pex4-interacting-regions of *Sc* Pex22 (aa 111-132) and *Oa* Pex22 (aa 99-120) aligned considerably more closely with the PEX4-interacting-residues of *At* PEX22 (aa 212-233) (∼0.8 Å RMSD over 22 Cα pairs) than the overall structures.

**FIGURE 5:**
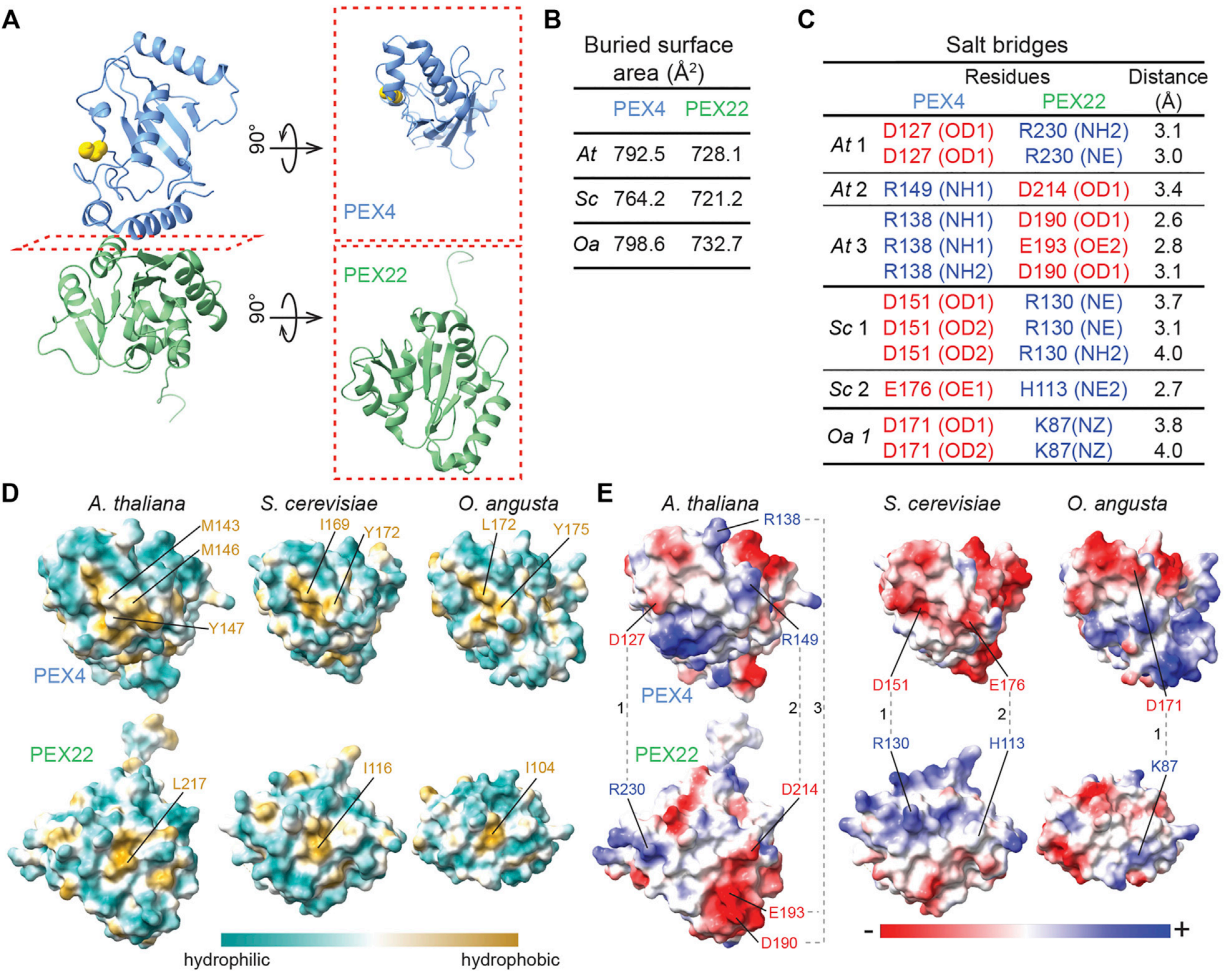
A central hydrophobic patch and complementary electrostatic surfaces mediate *Arabidopsis* PEX4-PEX22 interactions. **(A)**. Ribbon diagram of *A. thaliana* (*At*) PEX4-PEX22 (left) with subunits rotated 90° to allow a top view of the “bottom” of PEX4 and the “top” of PEX22 that form the interaction surface (right). **(B)**. Buried surface area at the interface between PEX4 and PEX22 deduced from the crystal structures using PDBePISA (Krissinel and Henrick, 2007; www.ebi.ac.uk/pdbe/prot_int/pistart.html). **(C)**. Salt bridges linking PEX4 and PEX22 predicted from the crystal structures using PDBePISA (Krissinel and Henrick, 2007; www.ebi.ac.uk/pdbe/prot_int/pistart.html). Positively charged residues are blue and negatively charged residues are red. Salt bridges are numbered as shown in panel **E** for the structures from each organism: *At, A. thaliana*; *Sc*, *S. cerevisiae*; and *Oa*, *O. angusta* Specific atoms involved in salt bridges are listed in parentheses (NE, epsilon nitrogen of arginine or histidine; NH, eta nitrogen of arginine; NZ, zeta nitrogen of lysine; OD, delta oxygen of aspartate; OE, epsilon oxygen of glutamate). **(D)**. Visualization of surface hydrophobicity of the interaction surfaces of PEX4 (top row) and PEX22 (bottom row) from *A. thaliana*, *S. cerevisiae*, and *O. angusta*. Structures are rotated as shown in the panel **A** right column. Residues in the central hydrophobic patches (gold) are labeled. **(E)**. Visualization of surface electrostatic potential of the interaction surfaces of PEX4 (top row) and PEX22 (bottom row) from *A. thaliana*, *S. cerevisiae*, and *O. angusta*. Structures are rotated as shown in the panel **A** right column. Residues involved in salt bridges **(B)** are labeled and connected with dashed lines. Complementary charge patterns are evident in each PEX4-PEX22 pair, despite the differences across species.

The PEX4-PEX22 interface is formed by interactions of the two C-terminal α -helices of PEX4 ( α -helix 3 and 4) with β-strand 3 and α -helix 5 in the middle region of PEX22^111−283^ (Figures 3, 5A; Supplementary Figure S1). Using PDBePISA, we quantified the buried surface area (BSA) at the interface of the PEX4-PEX22 complex (Figure 5B). PEX4 has a buried surface area of 793 Å^2^ and PEX22 has a buried surface area of 728 Å^2^. The interface comprises 8–9% of the surface area of each structure. We also used PDBePISA to identify three likely salt bridges at the interface of the PEX4-PEX22^111−283^ complex (Figures 5C,E) that are positioned to contribute to PEX4-PEX22 binding. We found that the residues forming these salt bridges are conserved in plant PEX4 and PEX22 orthologs from *Glycine max* and *Zea mays* (Figure 1A), suggesting that the mode of interaction may be conserved in higher plants.

### Electrostatic Interactions at the PEX4-PEX22 Interface Appear to Contribute to Binding Specificity

Despite the structural similarity of *A. thaliana* PEX4 and *S. cerevisiae* Pex4 (Figure 4B), genetic complementation experiments reveal that *At* PEX4 selectively binds *At* PEX22 and not *Sc* Pex22 and vice versa (Zolman et al., 2005). To examine PEX4-PEX22 binding specificity, we compared the hydrophobic (Figure 5D) and electrostatic (Figure 5E) characteristics of the interacting surfaces of PEX4 and PEX22 from *A. thaliana, S. cerevisiae*, and *O. angusta*. The backbones of the PEX4 proteins align at the PEX22 binding interface (Figure 4B), and all protein pairs have similarly sized interaction surfaces (720-800 Å^2^; Figure 5B). Moreover, the central hydrophobic pocket important for *S. cerevisiae* Pex4-Pex22 interaction (Williams et al., 2012) appears to be conserved among the three PEX4 proteins (Figures 1A, 5D). In contrast, the electrostatic properties of the surfaces are not well conserved (Figure 5E). For example, the positions of the salt bridges in the *A. thaliana* complex are not conserved in *S. cerevisiae* or *O. angusta* Pex4-Pex22 (Figures 5C,E), and residues corresponding to *A. thaliana* PEX4 R138 and R149, which are involved in salt bridges with PEX22, are uncharged in the yeast Pex4 proteins (Figures 1A, 5E).

**FIGURE 6:**
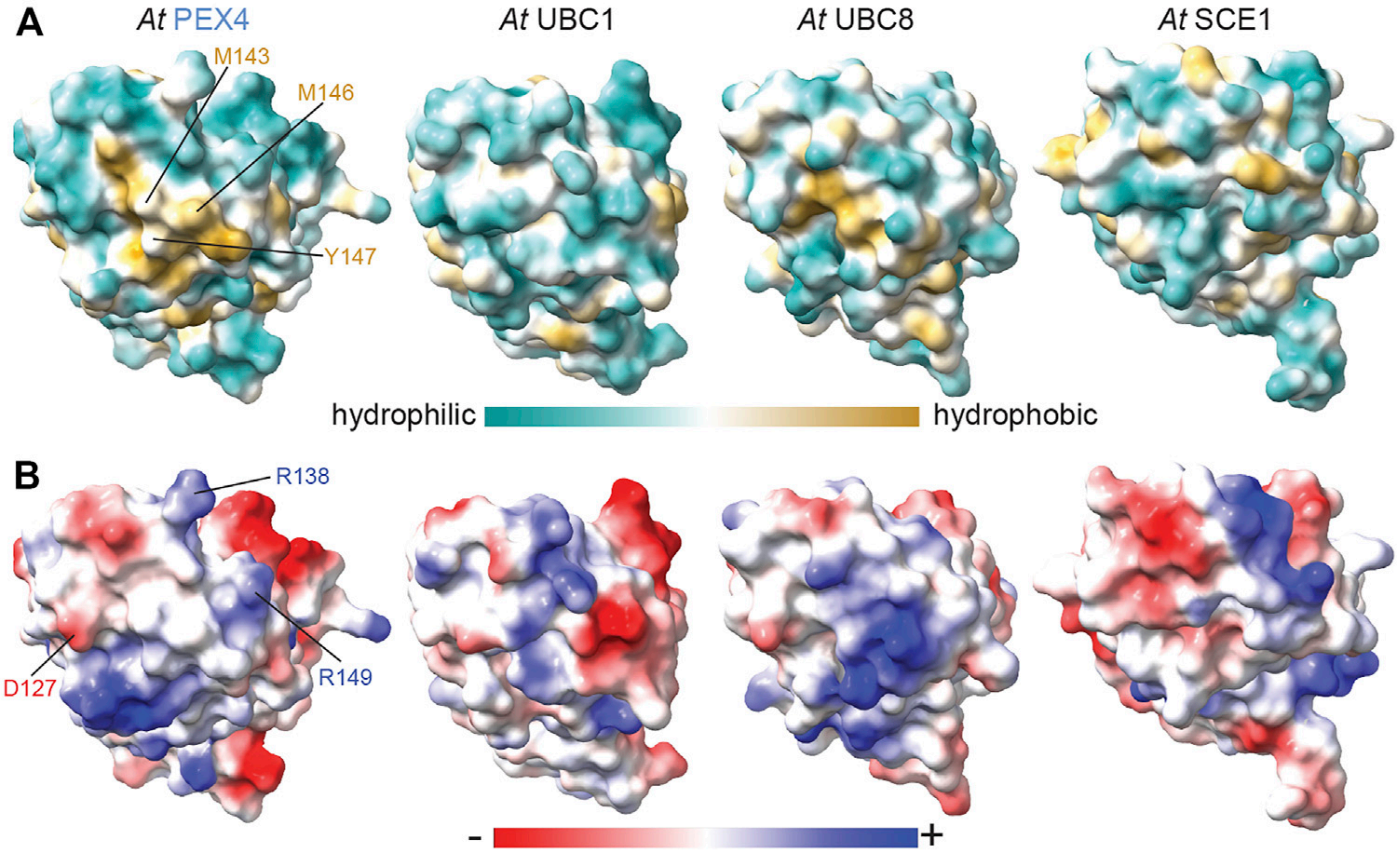
Hydrophobic and electrostatic properties of *Arabidopsis* ubiquitin- and sumo-conjugating enzyme surfaces provide insight into PEX4-PEX22 binding specificity. Surface hydrophobicity **(A)** and electrostatic **(B)** projections of *A. thaliana* (*At*) PEX4, UBC1 (PDB 2AAK), UBC8 (PDB 4X57), and SCE1 (PDB 6GV3). Structures were aligned to *At* PEX4 and are oriented as in Figure 5 to show the PEX22-interacting and analogous surfaces. PEX4 residues in the conserved hydrophobic patch implicated in PEX22 binding are labeled in panel **A**, and residues involved in salt bridges with PEX22 are labeled in panel **B**. The surfaces are quite distinct, consistent with the expected inability of PEX4 paralogs to bind PEX22.

Despite the overall similarity of PEX4 to other UBCs (Figures 1A, 4A), the peroxisome-defective phenotypes of *pex4* mutants (Zolman et al., 2005; Kao et al., 2016) imply that other *A. thaliana* UBCs do not substitute for PEX4 in binding to PEX22. To understand the basis for this selectivity, we compared the PEX22-interacting surface of PEX4 with the corresponding surfaces of similar *A. thaliana* enzymes with available structural information: UBC1, UBC8, and SCE1. This comparison revealed substantially different hydrophobicity patterns (Figure 6A) and a lack of charge conservation of at least one of the three PEX4 residues involved in salt bridges with PEX22 (Figures 1A, 6B) that are likely to preclude interaction with PEX22.

### PEX4 Builds K48-Linked Ubiquitin Chains and pex4-1 Displays Altered Ubiquitination Substrate Specificity

**FIGURE 7:**
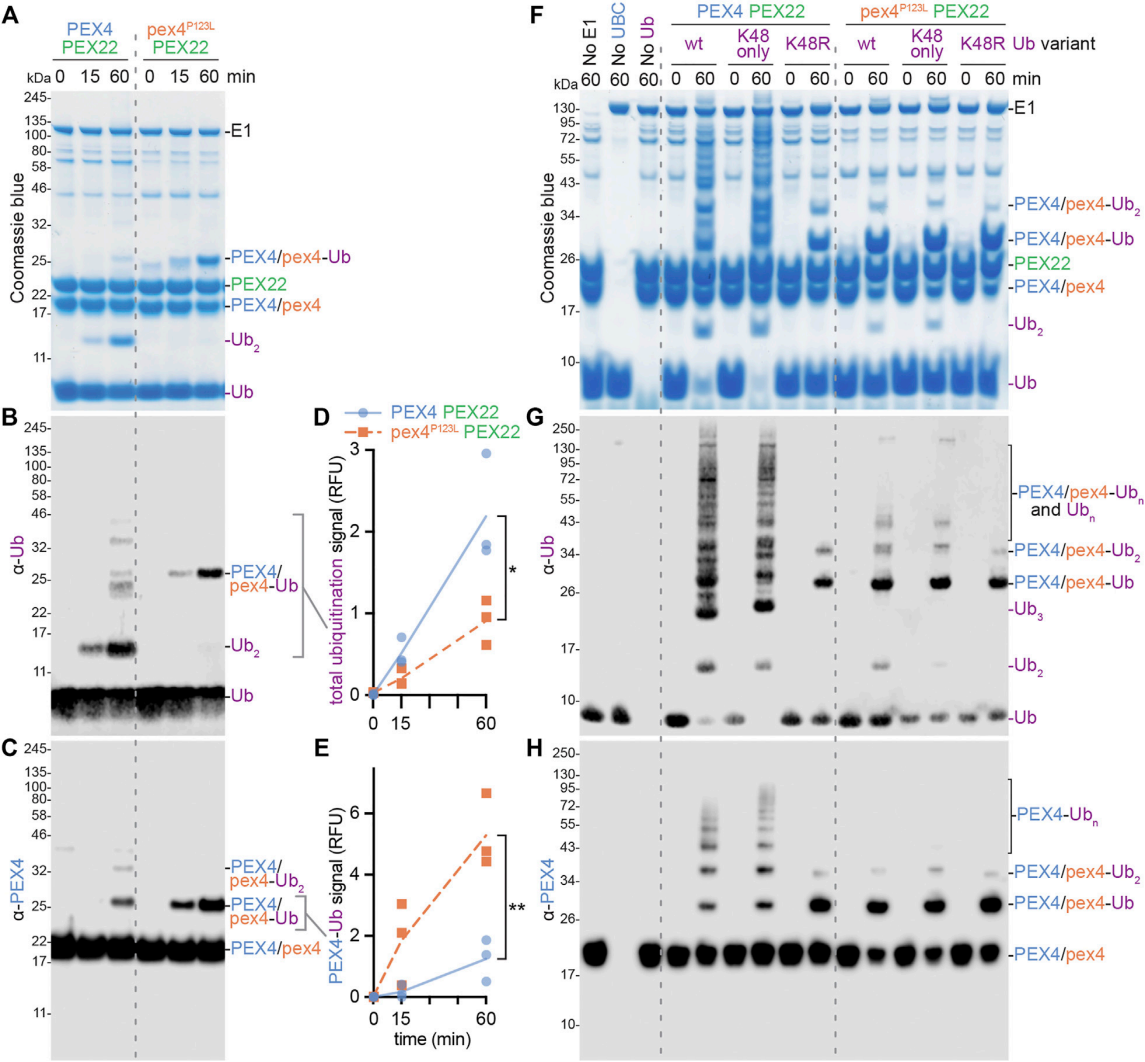
PEX4 builds K48-linked ubiquitin chains and pex4-1 (pex4^P123L^) displays altered ubiquitination activity *in vitro*. **(A–C)**. Ubiquitination assays containing recombinant human ubiquitin-activating enzyme (E1), *A. thaliana* UBC (either PEX4-PEX22^111−283^ or pex4^P123L^-PEX22^111−283^), and *A. thaliana* ubiquitin (Ub) were initiated by ATP addition and stopped at the indicated timepoints. The resulting samples were analyzed by SDS-PAGE followed by Coomassie staining **(A)** or immunoblotting with α-Ub **(B)** or α-PEX4 **(C)** antibodies. This experiment was conducted three times and representative results are shown. Ub, ubiquitin; Ub_2_, diubiquitin. **(D–E)**. Accumulation of various ubiquitinated proteins. Graphs display quantification of signals detected by the α-Ub **(D)** or α-PEX4 **(E)** antibodies in the indicated regions of the immunoblots **(B,C)**. Each point represents the quantification from a technical replicate at the indicated timepoint. The accumulation rate was calculated for each replicate, and the average rate is plotted. Significance was determined by two-tailed t-tests comparing the accumulation rates from pex4^P123L^-PEX22^111−283^ to PEX4-PEX22^111−283^. Brackets indicate significant differences between PEX4-PEX22^111−283^ and pex4^P123L^-PEX22^111−283^ rates (*p < 0.05; **p < 0.005). **(F**–**H)**. Ubiquitination assays as in panels **A**–**C** except that various ubiquitin variants were used: *A. thaliana* Ub (wt), human Ub with all lysine residues substituted with arginine except K48 (K48 only), or human Ub with K48 substituted with arginine (K48R). Samples were analyzed by SDS-PAGE followed by Coomassie staining **(F)** or immunoblotting with α-Ub **(G)** or α-PEX4 **(H)** antibodies.

Examining the PEX4 residue altered in the *pex4-1* missense allele revealed that the mutated P123 residue was positioned on the protein surface ∼6 Å from the PEX4 active site C90 residue (Figure 3); this proximity suggests that pex4-1 enzymatic activity might be impaired or modified. To directly examine the impacts of the pex4-1 mutation (P123L) on enzymatic function, we generated a construct to express His_6_-MBP-pex4^P123L^-PEX22^111−283^ in *E. coli*. Like the wild-type construct, the mutant fusion protein was soluble, and we purified pex4^P123L^-PEX22^111−283^ (Figure 2B) and compared *in vitro* self-ubiquitination activity of the two complexes. After incubating PEX4-PEX22^111−283^ or pex4^P123L^-PEX22^111−283^ with ubiquitin, ubiquitin-activating enzyme (E1), and ATP, we monitored self-ubiquitination using SDS-PAGE and Coomassie staining (Figure 7A) and immunoblotting with antibodies recognizing ubiquitin (Figure 7B) or PEX4 (Figure 7C).

We found that reactions using wild-type PEX4-PEX22^111−283^ accumulated a protein the size of diubiquitin and a ladder of ubiquitinated proteins of increasing molecular mass over time (Figures 7A,B,F,G), indicating that our assay successfully facilitated and detected *in vitro* ubiquitination. Immunoblotting revealed that one of the ubiquitinated proteins detected was PEX4 itself (Figures 7C,H), indicating that the wild-type complex was able to ubiquitinate not only ubiquitin but also PEX4. We found that pex4^P123L^-PEX22^111−283^ displayed somewhat reduced total ubiquitination activity (Figures 7B,D). Interestingly, pex4^P123L^-PEX22^111−283^ accumulated less diubiquitin (Figures 7A,B,F,G) and polyubiquitin ladder (Figure 7B) but instead accumulated increased levels of monoubiquitinated PEX4 (Figures 7A–C,E,F,H). Taken together, these data revealed that the pex4-1 mutation altered PEX4 substrate specificity without abolishing ubiquitination ability.

Ubiquitin contains seven lysine residues (and an N-terminus) that can be sites of ubiquitination; the resultant patterns of mono-, multi-, and poly-ubiquitination can target substrates for various fates (Yau and Rape, 2016). Because yeast Pex4-Pex22 builds K48-linked polyubiquitin chains on Pex4 (Groves et al., 2018), we also assessed PEX4-PEX22^111−283^ and pex4^P123L^-PEX22^111−283^ activity in the presence of ubiquitin (Ub) in which lysine 48 was replaced with arginine (Ub^K48R^) and ubiquitin in which all lysines other than K48 were replaced with arginine residues (Ub^K48only^). We found that ubiquitination patterns and levels were similar when wild-type PEX4-PEX22^111−283^ was provided with Ub or Ub^K48only^ (Figures 7F–H). In contrast, only mono- and di-ubiquitinated PEX4, and no polyubiquitin chains, were detected when PEX4-PEX22^111−283^ was provided with Ub^K48R^ (Figures 7F–H). These data indicate that PEX4-PEX22^111−283^ built polyubiquitin chains using K48 linkages. The persistence of di-ubiquitinated PEX4 when PEX4-PEX22^111−283^ was provided with Ub^K48R^ (Figures 7F–H) indicates that this di-ubiquitin is joined *via* a linkage other than K48, or that PEX4 is monoubiquitinated at two sites.

As with wild-type PEX4-PEX22^111−283^, the small amount of di-ubiquitin detected when pex4^P123L^-PEX22^111−283^ was incubated with Ub or Ub^K48only^ was abolished when pex4^P123L^-PEX22^111−283^ was incubated with Ub^K48R^. Interestingly, the ubiquitination pattern that resulted when PEX4-PEX22^111−283^ was provided with Ub^K48R^ resembled pex4^P123L^-PEX22^111−283^ provided with any of the ubiquitin variants (Figures 7G–I), reinforcing the conclusion that the pex4-1 mutation reduced the ability of PEX4 to build polyubiquitin chains.

### PEX4-PEX22 Binding Is Not Notably Impacted by the pex4-1 Mutation

**FIGURE 8:**
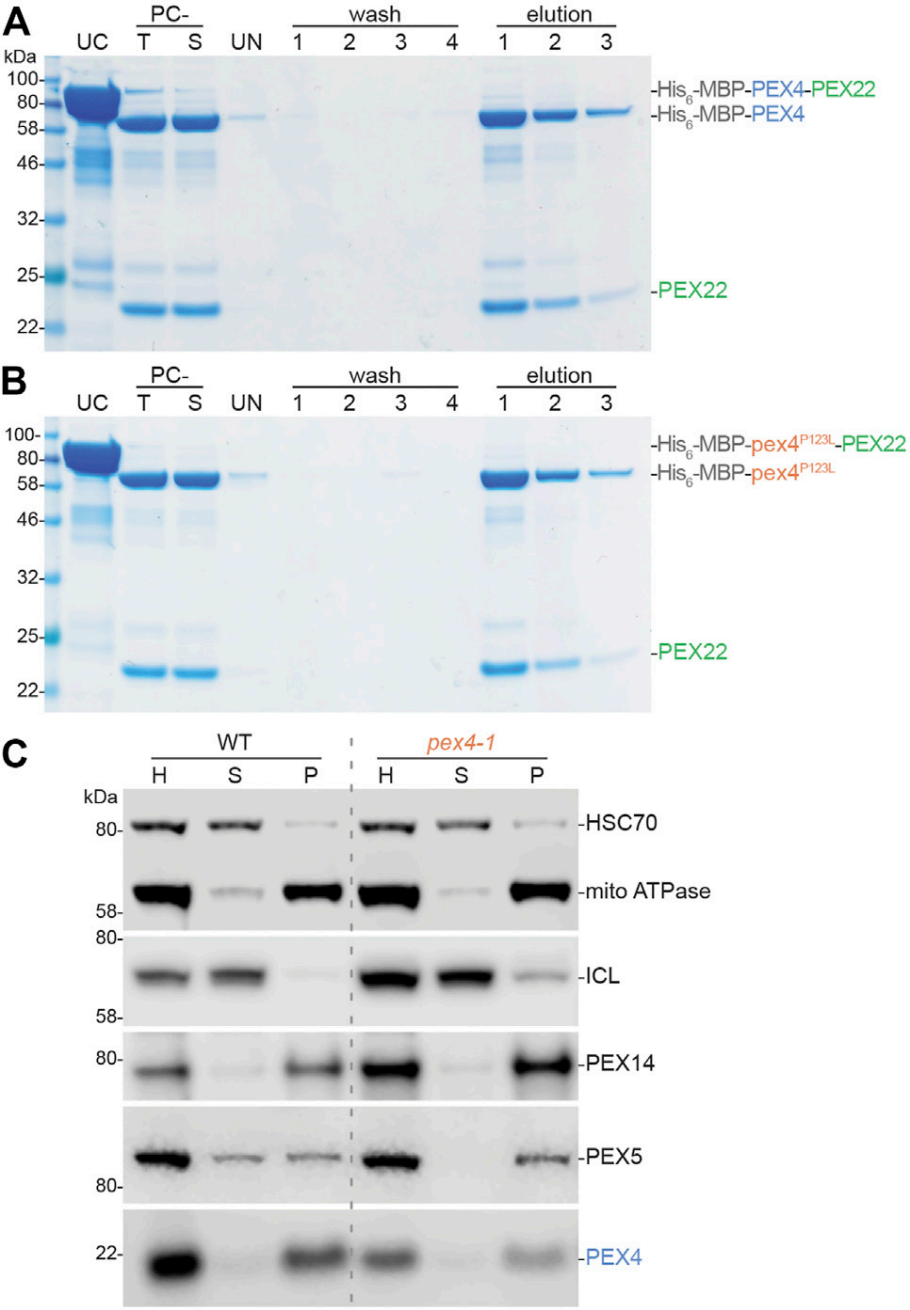
*Arabidopsis* PEX4 and pex4-1 proteins remain associated with PEX22. **A, B.** PEX4 and pex4-1 (pex4^P123L^) bind PEX22^111−283^ *in vitro*. Purified His_6_-MBP-PEX4-PEX22^111−283^ **(A)** or His_6_-MBP-pex4^P123L^-PEX22^111−283^ **(B)** fusion proteins (UC) were cleaved with PreScission protease, the PreScission-cleaved total (PC-T) reaction was centrifuged to remove insoluble protein, and the supernatant (PC-S) was incubated with Ni-NTA resin. After removing the unbound material (UN), the resin was washed four times in buffer without imidazole to remove unbound proteins (wash 1-4), then washed three times with imidazole to elute His-tagged proteins from the resin (elution 1-3). Equal portions of each fraction were analyzed *via* SDS-PAGE and Coomassie staining. PEX22^111−283^ co-purified similarly with wild-type MBP-PEX4 **(A)** and mutant MBP-pex4^P123L^ **(B)**. **(C)**. PEX4 and pex4-1 are membrane-associated (presumably via PEX22) in plants. Homogenates (H) from 6-day-old dark-grown seedlings were separated by centrifugation to yield soluble (supernatant, S) and insoluble (pellet, P) fractions. Samples were processed for immunoblotting and serially probed with antibodies recognizing HSC70 (soluble protein control), mitochondrial (mito) ATPase (membrane protein control), the peroxisome lumenal protein isocitrate lyase (ICL), and three peroxins (the membrane protein PEX14, the PEX5 receptor for lumenal proteins, and PEX4).

The Pex4-Pex22 complex is tightly bound in fungi, with dissociation constants of 1.9 nM in *O. angusta* (Ali et al., 2018) and 2.0 nM in *S. cerevisiae* (Williams et al., 2012). This tight association is likely explained by the extensive interactions revealed in the Pex4-Pex22 crystal structures (Figure 5; Williams et al., 2012; Ali et al., 2018). To assess binding of our recombinant PEX22^111−283^ to PEX4, we purified His_6_-MBP-PEX4-PEX22^111−283^, used the PreScission protease to release PEX22^111−283^, and then re-purified His_6_-MBP-PEX4 *via* nickel chromatography. SDS-PAGE (Figure 8A) revealed that PEX22^111−283^ remained bound to PEX4 throughout purification following cleavage, consistent with tight binding. We similarly tested pex4-1 in this assay and found that PEX22^111−283^ also co-purified with pex4-1 (Figure 8B).

To examine PEX4-PEX22 interactions in an endogenous context, we separated soluble and membrane-bound proteins from seedling extracts *via* centrifugation and examined peroxin localization by immunoblotting (Figure 8C). As expected, we found soluble (HSC70) and membrane (mitochondrial ATPase) proteins in the supernatant and pellet, respectively. The peroxisomal membrane peroxin PEX14 was in the pellet fraction, confirming that we had pelleted peroxisomal membranes. In contrast, the peroxisome lumenal protein isocitrate lyase was largely in the supernatant, indicating that the fractionation was not pelleting intact peroxisomes. PEX5 is recycled between the cytosol and the peroxisomal membrane and is found in both soluble and insoluble fractions (Ratzel et al., 2011). As previously reported (Ratzel et al., 2011; Kao and Bartel, 2015), PEX5 was more membrane-associated in *pex4-1* than in wild type (Figure 8C), presumably because PEX5 retrotranslocation from the peroxisomal membrane is reduced in *pex4-1*. Like PEX14, we found PEX4 largely in the pellet fraction in wild type (Figure 8C). This localization was unaltered in the *pex4-1* mutant (Figure 8C), implying that membrane association, presumably mediated by PEX22 interaction, is not dramatically impacted by the pex4-1 alteration.

## Discussion

To deepen our understanding of PEX4 function, PEX4-PEX22 interactions, and the potential molecular consequences of PEX4 mutations, we solved the structure of the *A. thaliana* PEX4-PEX22^111−283^ complex. To promote PEX4 folding, solubility, and enzymatic activity, we co-expressed PEX4 and PEX22 (without the N-terminal transmembrane domain) for structural and biochemical analyses (Figure 2). The *A. thaliana* PEX4 sequence is relatively conserved, with over 90% identity with other plant PEX4 orthologs, about 40% identity with yeast Pex4, and 35–40% identity with other *A. thaliana* UBCs (Figure 1A). As expected from this sequence conservation, the PEX4-PEX22^111−283^ crystal structure (PDB ID: 6XOD) revealed that PEX4 closely resembles other UBCs, including yeast Pex4 (Figures 4A,B). In contrast, PEX22 is much less conserved, and *A. thaliana* PEX22 shares only 55–69% amino acid sequence identity with PEX22 from other angiosperms, and no substantial identity with yeast Pex22 (Figure 1B). Despite this notable divergence, *A. thaliana* PEX22 and yeast Pex22 do share similar secondary structural elements that resemble a Rossmann fold (Figure 4C; Supplementary Figure S2).

When determining the PEX4 structure we were able to use *O. angusta* Pex4 for our initial molecular replacement model, but the PEX22 structure was completed using additional information from *ab initio* modeling. The use of modern modeling methods such as AlphaFold (Jumper et al., 2021) is expected to help in future structure determinations. In fact, the PEX22 structure was submitted as part of the CASP14 (Critical Assessment of Structure Prediction) competition (Ozden et al., 2021), and retrospective analysis showed that the best predicted models of PEX22 alone provided better initial molecular replacement solutions than did our PEX4 homology model.

PEX22 is more than a simple tether anchoring PEX4 to the peroxisomal membrane. For example, *O. angusta* Pex22 appears to activate Pex4 *via* allosteric active site remodeling (Groves et al., 2018). Upon binding to Pex22, an α -helix adjacent to the active-site cysteine in Pex4 relaxes to form a 3_10_ helix (Groves et al., 2018), a motif common in many UBCs (Streich and Lima, 2014). The 3_10_ helix is absent in *O. angusta* Pex4 crystallized without Pex22, and Pex4 without Pex22 is able to ubiquitinate Pex4 but unable to create polyubiquitin chains, hinting that this 3_10_ helix is necessary for full Pex4 activity (Groves et al., 2018). *A. thaliana* PEX4 in our PEX4-PEX22 structure forms the analogous 3_10_ helix near the active site (Figures 3–5), suggesting an active conformation. Like the yeast Pex4-Pex22 complex (Groves et al., 2018), the *A. thaliana* PEX4-PEX22 complex can build K48-linked polyubiquitin chains *in vitro* (Figure 7). We detected K48-linked polyubiquitin chains both on PEX4 (Figure 7H) and on ubiquitin (Figure 7G). Free ubiquitin chains were not detected in the yeast studies (Groves et al., 2018), and we did not exclude the possibility that these chains were built on ubiquitin linked *via* a thioester bond to the PEX4 active-site cysteine residue and released upon DTT treatment of the samples prior to electrophoresis.

Interestingly, we found that the *A. thaliana* pex4-1 enzyme is able to ubiquitinate pex4-1 but forms ubiquitin chains inefficiently even in the presence of PEX22 (Figure 7), reminiscent of yeast Pex4 in the absence of Pex22 (Groves et al., 2018). However, *A. thaliana* pex4-1 still binds tightly to PEX22 (Figure 8). Perhaps the pex4-1 P123L mutation causes a structural perturbation similar to that seen in yeast Pex4 in the absence of Pex22, but without preventing PEX22 binding.

**FIGURE 9:**
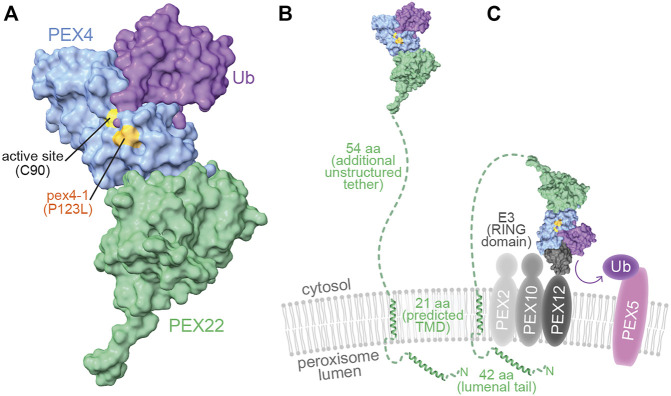
pex4-1 alters a residue near the PEX4 active site and PEX4 may reach distant peroxisomal membrane targets through an unstructured PEX22 tether. **(A)**. When charged, ubiquitin (purple) is predicted to dock onto PEX4 (blue) with the ubiquitin C-terminus nestled within the active site cleft, which is flanked by the residue mutated in pex4-1 (P123). A human UBC-Ub-RING domain co-structure (PDB ID 5FER) was aligned to the PEX4 chain of *At* PEX4-PEX22^111−283^ to show how PEX4 might interact with ubiquitin. **(B)**. The model of PEX4-PEX22-Ub from panel **A** displayed to approximate scale with the peroxisomal membrane. The N-terminal 117 amino acid residues (aa) of PEX22 (dashed green line and ribbon diagrams) were not part of the solved structure and include a peroxisomal lumenal tail predicted to include an alpha helix, a predicted α-helical transmembrane domain (TMD) that anchors PEX22 in the peroxisomal membrane, and an unstructured tether that links the TMD to the PEX4-binding domain of PEX22. The TMD and lumenal ribbon structures were predicted using AlphaFold Monomer v2.0 (Jumper et al., 2021; Varadi et al., 2022). **(C)**. PEX4-PEX22-Ub is predicted to bind the RING domain of the E3 ubiquitin-protein ligases PEX2, PEX10, or PEX12 (charcoal) in the peroxisomal membrane to ubiquitinate substrates including PEX5 (pink). The UBC of a human UBC-Ub-RING domain co-structure (PDB ID 5FER) was aligned to the PEX4 chain of *At* PEX4-PEX22^111−283^ to show how PEX4 (blue) might interact with ubiquitin (purple) and a RING domain (charcoal) to promote substrate (e.g., PEX5) ubiquitination.

Alternatively, the pex4-1 P123L mutation could alter catalysis more directly. P123 is positioned on a loop near the active site cleft (Figures 3, 9A), and pex4-1 alters the residue immediately following the “gateway residue” implicated in modulating active site access (reviewed in Stewart et al., 2016). This residue is often an aspartate or a serine (e.g., PEX4 S122 in Figure 1A), and phosphorylation of a serine at this position may modulate the activity of some human UBCs (reviewed in Stewart et al., 2016). Although S122 adjacent to the pex4-1 mutation is conserved in various PEX4 enzymes (Figure 1A), phosphorylation of *A. thaliana* PEX4 has not been reported in phosphoproteomic studies compiled in the PhosPhAt database (Heazlewood et al., 2008). Moreover, the altered *in vitro* pex4-1 activity that we observed with protein purified from *E. coli* indicates that the pex4-1 mutation changes enzymatic activity even in the absence of phosphorylation. The proximity of P123 to the active site cysteine (Figures 3, 9A), along with the modified *in vitro* activity of the pex4-1 enzyme (Figure 7), highlights the importance of this loop for UBC activity.

Our crystal structure also provides insight into the expected specificity of the PEX4-PEX22 interaction. Of the 37 predicted or confirmed UBCs in *A. thaliana* (Bachmair et al., 2001; Kraft et al., 2005), only PEX22 is expected to bind PEX4. Comparing the PEX22-binding surface of PEX4 to several other UBCs revealed differences in both hydrophobicity and electrostatics that could contribute to specificity (Figure 6). However, we did not directly examine PEX22 interactions with other *A. thaliana* UBCs, and isolation and characterization of a *pex4* null allele will be necessary to definitively determine whether any *A. thaliana* UBCs can substitute for PEX4 *in vivo*. Like yeast and plants, *Trypanosoma brucei* utilizes a PEX4-PEX22 system to ubiquitinate PEX5 (Gualdrón-López et al., 2013). Interestingly, residual lumenal protein import and ubiquitinated PEX5 are observed in a *T. brucei pex4* null mutant, implying that another UBC can partially substitute in the absence of PEX4 in this protozoan (Gualdrón-López et al., 2013). Whether this residual function requires *T. brucei* PEX22 has not been reported.

Although the PEX4 and PEX22 residues involved in salt bridges are conserved in plants (Figure 1), the details of the interaction have diverged during evolution. The *S. cerevisiae* and *O. angusta* Pex4-Pex22 interaction surfaces show substantially different electrostatic patterns (Figures 1, 5E), including differently positioned salt bridges (Figures 5C,E). Indeed, although expression of *A. thaliana PEX4* is unable to rescue a *S. cerevisiae pex4* mutant, and *At PEX22* is unable to rescue a *Sc pex22* mutant, expressing *At PEX4* and *At PEX22* together rescue either *Sc pex4* or *pex22* mutants (Zolman et al., 2005). These experiments reveal that *At* PEX4 is active in yeast when bound to *At* PEX22 and imply that *At* PEX4 does not bind to *Sc* Pex22 and that *Sc* Pex4 does not bind to *At* PEX22.

The PEX4-PEX22 interface covers about 800 Å^2^, which is smaller than many heterodimer interfaces with nM dissociation constants (Chen et al., 2013). The strong affinity (∼2 nM dissociation constants) of the yeast Pex4-Pex22 complexes (Williams et al., 2012; Groves et al., 2018) is likely shared by the *A. thaliana* PEX4-PEX22 complex; we did not find non-denaturing conditions that dissociated PEX4 and PEX22 (Figure 8 and data not shown). It is likely that multiple features contribute to this tight binding. The hydrophobic residue Y172 at the Pex22-interacting surface of *Sc* Pex4 is necessary for *Sc* Pex4-Pex22 binding (Williams et al., 2012). The central hydrophobic patches on the interacting surfaces of Pex4 and Pex22 appear to be conserved across *A. thaliana*, *S. cerevisiae*, and *O. angusta* (Figure 5D), suggesting that the central hydrophobic region is important for PEX4-PEX22 binding. Taken together, these data imply that the tight binding of PEX4-PEX22 is dominated by conserved hydrophobic interactions at the center of the interaction surface, while binding specificity is controlled by flanking non-conserved salt bridges.

As a UBC, PEX4 is expected to have multiple interacting partners beyond PEX22, including ubiquitin, the ubiquitin-activating enzyme, and the RING-type ubiquitin-protein ligases, PEX2, PEX10, and PEX12. Structural studies of other UBCs have revealed that the ubiquitin-activating enzyme and the RING domains of ubiquitin protein ligases bind to overlapping surfaces on the active-site face and top of the UBC (Olsen and Lima, 2013; Koliopoulos et al., 2016). Thus, PEX4 presumably alternates between binding the ubiquitin-activating enzyme to receive ubiquitin and binding the RING peroxins to ubiquitinate substrates. The position of the PEX22-binding surface on the bottom of PEX4 (Figures 3, 9) is distinct from the predicted ubiquitin-activating enzyme (not shown) and RING interaction surfaces (Figure 9C). This use of non-overlapping interaction surfaces, along with the tight binding of PEX4 to PEX22 (Figure 8) implies that PEX4 can cycle through ubiquitin loading and delivery without releasing PEX22.

In addition to interactions with the other enzymes in the ubiquitination cascade, several UBCs non-covalently bind ubiquitin, ubiquitin chains, or ubiquitin-like proteins on the “backside” of the enzyme (reviewed in Stewart et al., 2016). This binding can provide allosteric regulation to increase chain-building processivity. Moreover, Ubc7, which acts in ER-associated protein degradation, binds the Cue1 ER tether *via* the backside (reviewed in Stewart et al., 2016). Cue1 binding of the Ubc7 backside both tethers Ubc7 to the ER and increases Ubc7-RING domain binding affinity (Metzger et al., 2013). Similarly, membrane-anchored ubiquitin fold (MUB) proteins tether UBCs to the plasma membrane *via* backside binding (Lu et al., 2016). Unlike these other tethered UBCs, the backside of PEX4 appears to remain free for additional interactions even when PEX4 is tethered to the peroxisomal membrane *via* PEX22 binding (Figure 3), providing additional opportunities for allosteric regulation.

The PEX4-PEX22 structure illuminates potential molecular consequences of the *pex4-1* missense mutation. The mutated P123 residue is positioned on the protein surface ∼6 Å from the PEX4 active site C90 residue (Figures 3, 9A). To visualize this pex4-1 alteration and the PEX4-PEX22 interaction surface in relation to other expected PEX4 binding partners, we modeled possible PEX4 interactions with ubiquitin (Figure 9A) and the RING domain of a ubiquitin-protein ligase (Figure 9C). This modeling took advantage of the similarity of the PEX4 structure to other UBCs, including a human UBC that has been co-crystalized with ubiquitin and a RING domain (Koliopoulos et al., 2016; PDB 5FER). Our model predicts that the residue altered by the pex4-1 mutation (P123) borders the active site cleft that holds the C-terminal tail of ubiquitin (Figure 9A) and is consistent with the altered *in vitro* ubiquitination selectivity of pex4^P123L^-PEX22^111−283^ (Figure 7).

The availability of enzymatically active *A. thaliana* PEX4 and pex4-1 (Figure 7) will enable future biochemical and structural studies with PEX4, the RING peroxins, and their various substrates. The three RING peroxins form a complex (El Magraoui et al., 2012) and act non-redundantly to maintain peroxisome function. In yeast, Pex12 works with Pex4 to monoubiquitinate Pex5 for recycling, Pex2 works with the cytosolic Ubc4 to polyubiquitinate Pex5 for degradation (Kragt et al., 2005; Williams et al., 2008; Platta et al., 2009), and Pex10 enhances both activities (El Magraoui et al., 2012). Interestingly, *Sc* Ubc4 targets Pex5 lysine residues, whereas *Sc* Pex4 ubiquitinates Pex5 on a conserved cysteine residue *in vivo* (Williams et al., 2007); the analogous PEX5 cysteine residue is also ubiquitinated in mammalian cells (Carvalho et al., 2007). Although PEX5 ubiquitination has not been directly demonstrated in plants, the relevant cysteine residue is conserved, and accumulating indirect evidence supports the hypothesis that PEX5 ubiquitination is necessary for PEX5 recycling in *A. thaliana*. *At* PEX5 is more membrane associated in *pex4* mutants than in wild type (Figure 8C; Ratzel et al., 2011; Kao and Bartel, 2015), suggesting that PEX4 is involved in the ubiquitination needed to remove PEX5 from the peroxisomal membrane. Moreover, *At* PEX5 levels are decreased in most *pex6* mutants (Zolman and Bartel, 2004; Gonzalez et al., 2017, 2018), which are defective in an ATPase implicated in retrotranslocating ubiquitinated PEX5 from the membrane. In addition, PEX5 levels are restored in *pex6-1* double mutants with *pex4-1* (Ratzel et al., 2011) or *pex2-1* (Burkhart et al., 2014). These data implicate *At* PEX4 in the PEX5 degradation that ensues when PEX5 recycling is slowed. Together, these studies suggest that PEX4 ubiquitinates PEX5 for both recycling and degradation in plants, and it will be interesting to learn if this ubiquitination can be reconstituted *in vitro*. The RING peroxins are also implicated in ubiquitination and degradation of other proteins in the peroxisomal membrane (Williams and van der Klei, 2013; Chen et al., 2018). The *A. thaliana* RING peroxins are essential for embryogenesis (Schumann et al., 2003; Sparkes et al., 2003; Fan et al., 2005; Prestele et al., 2010) and RING domains of *At* PEX2, PEX10, and PEX12 display *in vitro* monoubiquitination activity when paired with human UBCH5b/c (Kaur et al., 2012). Future studies may reveal how RING peroxin activity is impacted by *A. thaliana* PEX4 versus cytosolic UBCs.

Although not part of our crystal structure, a 62-amino acid tether links the N-terminus of α-helix 1 of the structured region of PEX22 to the C-terminal end of the *At* PEX22 predicted transmembrane domain (Figure 9B). This tether is poorly conserved (Figure 1B) and lacks confidently predicted secondary structure (Jumper et al., 2021). The corresponding regions separating the N-termini of the first yeast Pex22 structural elements (β-strand 1; Supplementary Figure S2) from the C-termini of the predicted transmembrane domains also lack predicted secondary structure but are considerably shorter (21-25 aa; Figure 1B). An extended conformation and lack of secondary structure in this linker region would allow *At* PEX4 to reach relatively distant targets (∼200 Å) on the peroxisomal membrane (Figure 9C). It will be interesting to learn the full repertoire of PEX4 substrates, and whether the lengthening of this tether in plants (Figure 1B) has functional significance.

## Data Availability

The original contributions presented in the study are included in the article/Supplementary Material, further inquiries can be directed to the corresponding author.

## Abbreviations

aa, amino acid residues; At, *Arabidopsis thaliana;* BSA, buried surface area; DTT, dithiothreitol; ER, endoplasmic reticulum; ICL, isocitrate lyase; LB, Luria broth; mito, mitochondrial; Oa, *Ogataea angusta;* PDB, Protein Data Bank; PEX, peroxin; PN, plant nutrient; PTS, peroxisome targeting signal; RING, really interesting new gene; RMSD, root-mean-square deviation; RT, room temperature; RWD, domain found in RING fingers, WD-domain-containing-proteins, and DEAD-like helicases; Sc, *Saccharomyces cerevisiae;* SCE, SUMO conjugating enzyme; TB, terrific broth; TCEP, Tris(2-carboxyethyl)phosphine hydrochloride; TMD, transmembrane domain; Ub, ubiquitin; UBC, ubiquitin conjugating enzyme; UEV, ubiquitin E2 variant; IPTG, β-D-1-thiogalactopyranoside.
