# Supplementary material for: The Structure of the Arabidopsis PEX4-PEX22 Peroxin Complex—Insights Into Ubiquitination at the Peroxisomal Membrane
Source: Front Cell Dev Biol. 2022 Feb 18;10:838923. doi: 10.3389/fcell.2022.838923 (PMC8922245; doi:10.3389/fcell.2022.838923)
Supplement: Supplementary file 1 [file DataSheet1.PDF]

Supplementary Material

Supplemental Table 1. PCR primers used for plasmid construction

Primer name	Primer sequence*	Purpose
pET-F<br>pET-R	GAACAGTTCTTTGGAAGTGTTTAAAATATTGGAAGTGGATAACGG<br>CTTGATGCCTGCATGGATTGGAAGTACAGGTTTTCC	amplifying pET expression vector
PEX4-F<br>PEX4-R	GGAAAACCTGTACTTCCAATCCATGCAGGCATCAAGAGC<br>GTCCTTGAAACAGCACTTCCAGTCCTTTCTTAGGCATAGCGG	amplifying *PEX4* cDNA for PEX4-PEX22 expression vector
PEX22-F<br>PEX22-R	GTGCTGTTTCAAGGACCAGCGGTGCAAGATGTTGTTGATC<br>CCGTTATCCACTTCCAATATTTTAAACACTTCCAAAGAACTGTTC	amplifying *PEX22* cDNA for PEX4-PEX22 expression vector
pex4-1-R*<br>pex4-1-PEX22-F*	GATTCCCTGAGTCGCAGTTAAGAaGACTGTCCGGCTCAG<br>TCATCCTGAGCCGGACAGTCtTCTTAACTGCGACT	generating pex4-1-PEX22 expression vector

*Nucleotides altered to introduce the *pex4-1* mutation are in lower case.

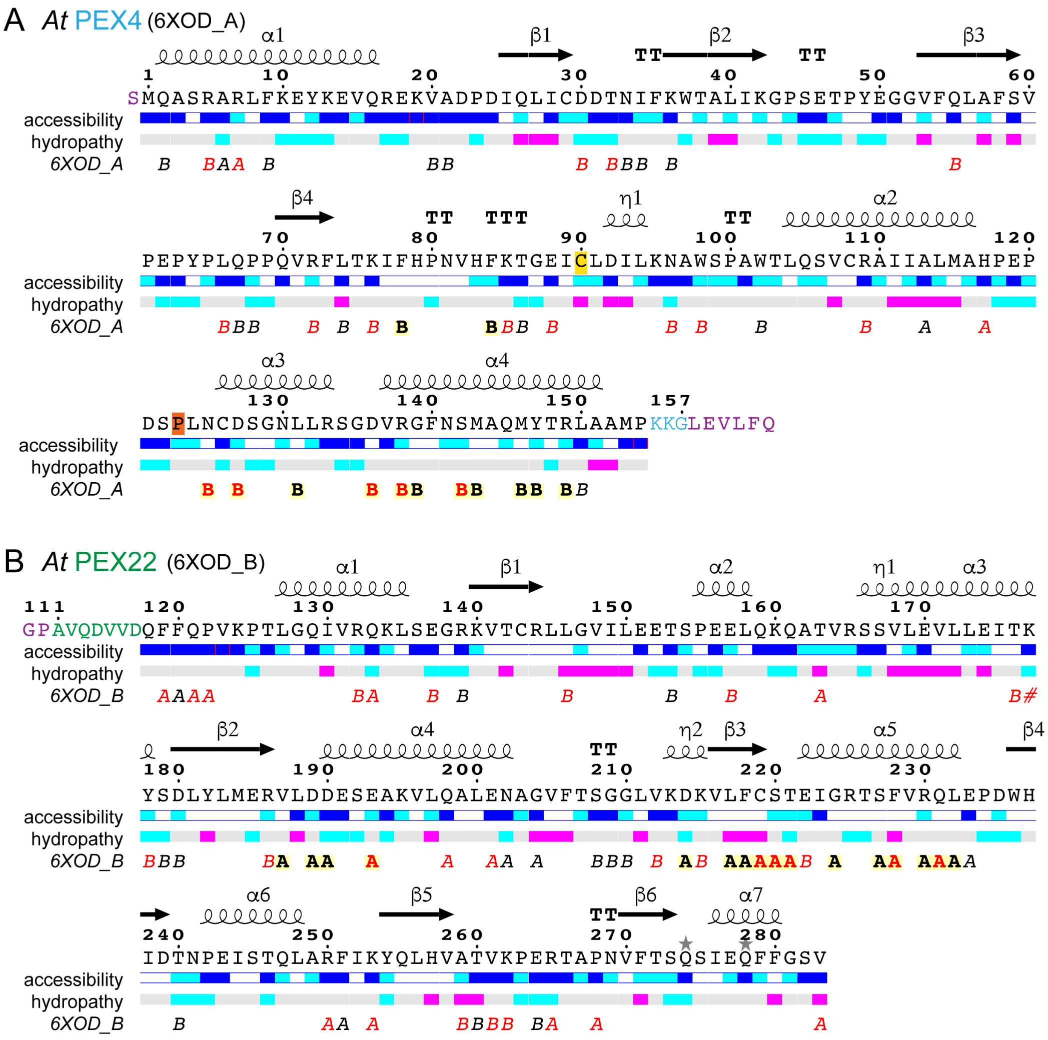

Supplemental Figure 1. Structural elements of *Arabidopsis* PEX4 (**A**) and PEX22 (**B**) derived from the crystal structure (6XOD) using the ENDscript 2 program (https://endscript.ibcp.fr; Robert and Gouet, 2014).

Elements of secondary structure are illustrated above the sequence. Structured residues are in black font; disordered residues include PEX4$^{155\text{-}157}$ (blue), PEX22$^{111\text{-}117}$ (green), and residues added to allow protease cleavage (purple). The PEX4 active site (C90) and the residue altered in pex4-1 (P123) are highlighted in yellow and orange, respectively. In the “accessibility” rows, accessible residues are blue, intermediate residues are cyan, and buried residues are white. In the “hydropathy” rows, hydrophobic residues are pink, intermediate residues are gray, and hydrophilic residues are cyan. In the “6XOD” rows, red letters identify <3.2 Å contacts and black letters identify contacts between 3.2 and 5.0 Å. Bold letters with a yellow background designate non-crystallographic contacts between PEX4 and PEX22. Gray stars mark residues with alternate conformations.

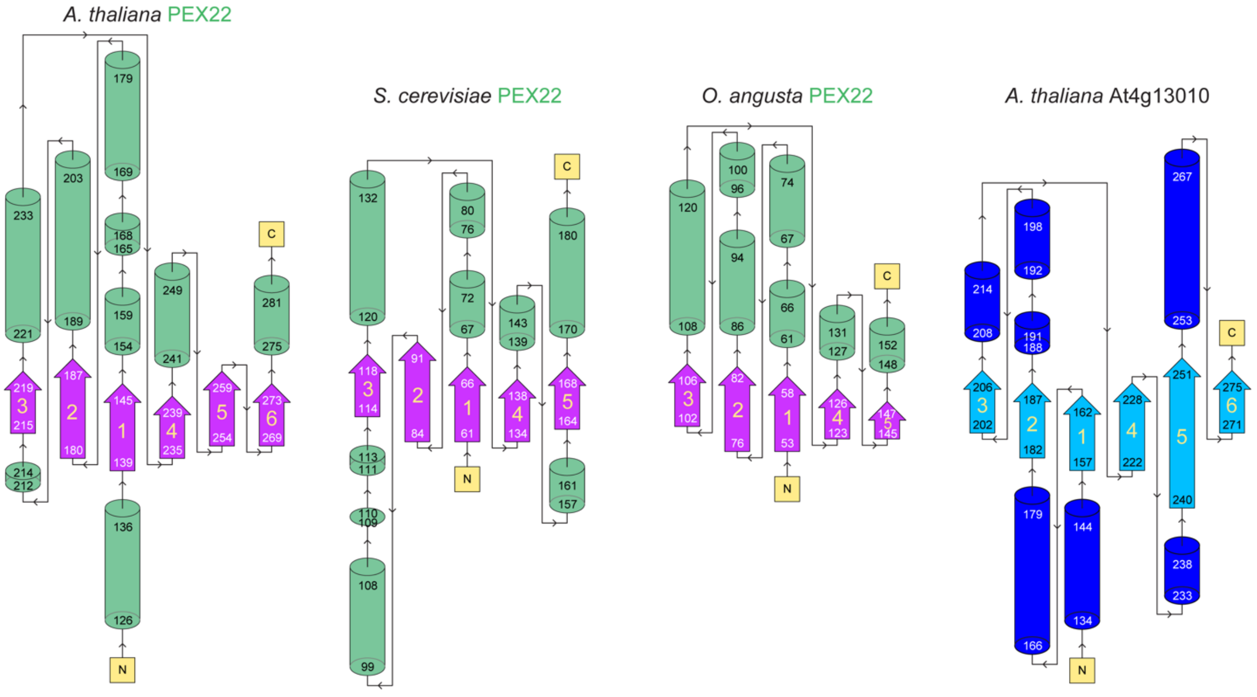

Supplemental Figure 2. *A. thaliana* PEX22 topology plot depicts a Rossmann fold similar to yeast Pex22.

Topology plots generated using PDBsum (Laskowski et al., 2018) of *At* PEX22 (PDB 6XOD), *Sc* Pex22 (PDB 2Y9M), and *Oa* Pex22 (PDB 5NKZ) are shown. An *A. thaliana* protein (At4g13010; PDB 5A3V) that adopts a Rossmann fold is shown in blue for comparison. α-Helices are depicted as cylinders, and β-strands are depicted as broad arrows and numbered with yellow text. Numbers in white and black text indicate amino acid residues at the ends of structural elements. N- and C-termini are indicated in yellow boxes.
